# A Sub‐1 Nm Cluster Chains‐enhanced Poly(ethylene oxide) Electrolyte for an All‐solid‐State Lithium Metal Battery with a Long Cycling Lifespan

**DOI:** 10.1002/advs.202516696

**Published:** 2025-11-28

**Authors:** Shanshan Song, Fei He, Yijun Gao, Yumeng Zhang, Junming Zhang, Tongjiao Yin, Qiqi Sun, Zhiliang Liu, Piaoping Yang

**Affiliations:** ^1^ Key Laboratory of Superlight Materials and Surface Technology Ministry of Education College of Materials Science and Chemical Engineering Harbin Engineering University Harbin 150001 P. R. China

**Keywords:** all‐solid‐state lithium batteries, cationic vacancy, inorganic cluster chain, Li^+^ transport

## Abstract

Flexible composite polymer electrolytes (CPEs) are promising candidates for high‐energy all‐solid‐state lithium metal batteries, owing to their superior processability, excellent electrochemical performance, and enhanced safety. Nevertheless, conventional CPEs face challenges such as lithium‐ion blocking and aggregation from inert fillers, hindering ion transport. To address these issues, highly dispersed materials need optimization in polymer matrices. In this study, a new strategy is introduced to introduce sub‐1 nm inorganic cluster chains into poly(ethylene oxide) based electrolytes. These ultra‐thin highly dispersed cluster chains eliminated the Li blocking region and aggregation‐induced barrier, formed a 3D network, and realized the efficient conductivity of Li^+^. By engineering La vacancies into the cluster chains of Ta‐doped LaOOH crystals, low‐energy migration pathways are created. This optimized electrolyte achieves the ionic conductivity of 0.65 mS cm^−^^1^ at 60 °C and the lithium transference number of 0.47. Electrochemical testing shows outstanding stability: Li||LTPE||Li symmetric cells maintain stable Li plating and stripping for 2000 h, and LiFePO_4_||LTPE||Li cells achieve 138.5 mAh g^−1^ with 93.4% capacity retention after 2000 cycles at 2C. Furthermore, the pioneering application of in situ conductive atomic force microscopy enables unprecedented characterization of temperature‐dependent morphological evolution and heterogeneous ion transport dynamics in solid electrolytes.

## Graphical abstract

Sub‐1 nm inorganic cluster chain with cation vacancies optimize PEO electrolytes by eliminating Li^+^ migration barriers and constructing 3D ion transport networks, while in situ c‐AFM reveals dynamic transport mechanisms, enabling high‐performance all‐solid‐state batteries.

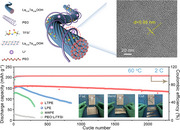

## 1 Introduction

Lithium metal batteries (LMBs) offer high theoretical specific capacity (3860 mAh g^−1^) and extremely low oxidation‐reduction potential (−3.04 V vs SHE), driving demand beyond conventional Li‐ion batteries.^[^ ^1^ ^]^ However, organic liquid electrolytes pose flammability and leakage risks. Therefore, the development of high‐performance composite polymer electrolytes (CPEs) is crucial for next‐generation all‐solid‐state lithium metal batteries (ASSLMBs), promising higher energy density and enhanced safety.^[^ ^2^ ^]^ While CPEs show promising potential, conventional polyethylene oxide (PEO)‐based CPEs still exhibit several inherent drawbacks, particularly due to low ion conductivity resulting from polymer crystallization, interface instability induced by filler aggregation, and the formation of discontinuous ion transport pathways.^[^ ^3^, ^4^ ^]^

The ion conduction of PEO‐based electrolytes is predominantly confined to their amorphous region. However, the inherent crystallinity of PEO limits the volume fraction of these conductive domains, thereby severely restricting the formation of continuous ion transport pathways.^[^ ^5^ ^]^ In order to improve the ion conductivity of PEO, various methods have been studied, including copolymerization,^[^ ^6^, ^7^ ^]^ cross‐linking,^[^ ^8^, ^9^ ^]^ and branching^[^ ^10^ ^]^ to reduce the crystallinity of PEO. On the other hand, research mainly focuses on suppressing the crystallization of PEO and creating additional ion transport channels by doping fillers or optimizing the kinetics of polymer chains to significantly improve ion conductivity. Therefore, smaller‐sized inert fillers reduce Li⁺‐insulating regions, increase the density of surface active sites, and improve organic/inorganic interfacial contact, thereby facilitating rapid Li⁺ transport.^[^ ^11^ ^]^ The Mustarelli group reported that CPEs with the pegylated SiO_2_ nanoparticles (diameter 30 nm) showed ionic conductivity of 0.1 mS cm^−1^ at 25 °C.^[^ ^12^ ^]^ A critical challenge, however, is the aggregation of nanoscale fillers driven by their high surface energy, which can block percolation pathways at the polymer‐filler interface. Therefore, strategic dispersion control and precise particle size optimization are imperative to construct interconnected ion‐conduction networks for superior Li⁺ mobility.

Defect engineering of inorganic fillers has emerged as a pivotal strategy for optimizing Li^+^ diffusion pathways in PEO‐based solid‐state electrolytes. Targeted defect engineering‐particularly vacancy generation via doping, non‐equilibrium plasma processing, or radiation‐induced lattice disorder—precisely modulates electrochemical potential gradients and crystallographic configurations.^[^ ^13^, ^14^, ^15^, ^16^, ^17^, ^18^, ^19^ ^]^ The defect‐rich surfaces can significantly enhance interfacial ion dynamics by providing abundant adsorption/desorption sites for Li^+^.^[^ ^20^ ^]^ Moreover, specific defect configurations may establish low‐energy‐barrier interstitial channels that facilitate Li⁺ migration with reduced activation energy, as demonstrated by Density Functional Theory (DFT) calculations. Experimental studies reveal that oxygen vacancies not only strengthen the interfacial interaction between TiO_2_ and PEO chains through enhanced Lewis acid‐base interactions, but also create additional percolation pathways for Li⁺ transport (5.8 × 10^−4^ S cm^−1^ at 50 °C).^[^ ^21^ ^]^ Current research predominantly focuses on anion vacancy manipulation generation, while systematic investigations into cation vacancy engineering remain scarce. Xu et al.'s study demonstrates that Co‐doping of Ta and Ga stabilizes the cubic phase of LLZO (Li_7_La_3_Zr_2_O_12_), effectively preventing its transition to the low‐ionic‐conductivity tetragonal phase at lower temperatures.^[^ ^22^ ^]^ This stabilization maintains a robust framework that facilitates Li^+^ transport. The combined presence of Ta^5+^ and Ga^3+^ synergistically promotes Li^+^ migration, an effect further aided by the Coulombic repulsion from Ga^3+^. Additionally, doping optimizes the lattice parameters, thereby broadening the Li^+^ transport channels Yao et al.'s research highlights that in the LaCl_3_ system, Ta doping induces La vacancies within the lattice.^[^ ^23^ ^]^ These vacancies connect the originally isolated 1D ionic channels, forming a 3D Li^+^ migration network. This mechanism overcomes the inherent limitations of ionic transport in traditional chloride‐based solid‐state electrolytes. This approach effectively addresses key challenges faced by conventional solid‐state electrolytes, including low ionic conductivity and interface instability. These findings underscore the significant potential of Ta doping for enhancing the performance of solid‐state electrolytes. Cationic vacancy engineering has emerged as a powerful strategy for enhancing ionic conductivity in inorganic materials.^[^ ^24^ ^]^ This approach involves the deliberate introduction of vacancies within the cation sublattice of inorganic fillers, creating additional migration sites for charge carriers such as Li⁺. This modification optimizes ionic transport pathways, reduces the energy barrier for ionic migration, and consequently improves overall ionic conductivity. Techniques such as elemental doping and exploiting high‐entropy effects enable precise control over vacancy concentration. This control mitigates transport pathway blockage caused by vacancy aggregation and maximizes ionic transport efficiency.^[^ ^25^ ^]^ Similarly, in the work of He et al., fillers containing cationic vacancies were incorporated into polymer composite solid electrolytes.^[^ ^26^ ^]^ Their findings indicate that the enhanced Li⁺ conductivity is attributed to two key mechanisms: The vacancy‐induced porous architecture provides rapid ion transport channels, facilitating an enhanced ion‐hopping mechanism. This interaction promotes the dissociation of lithium salts, thereby increasing the population of freely mobile Li⁺. It is necessary to explore the influence of cationic vacancy engineering on CPEs. Advanced characterization techniques, including electrochemical impedance spectroscopy (EIS), solid‐state nuclear magnetic resonance (NMR), and electrochemical strain microscopy (ESM) have been widely employed to probe Li⁺ transport mechanisms.^[^ ^27^ ^]^ However, these methods primarily provide ensemble‐averaged macroscopic measurements or indirect correlations of ionic motion, failing to directly visualize dynamic Li⁺ migration at atomic‐scale defects. Therefore, an intuitive method is urgently needed to monitor the migration of lithium ions.

In this work, we present a comprehensive and innovative multiscale design paradigm to surmount these formidable challenges. At the sub‐nanoscale level, we introduce Ta‐doped LaOOH cluster chains (La_x_Ta_y_OOH (y = 0.02, 0.04, 0.06, and 0.08) into the PEO matrix. Leveraging the quantum confinement effect to improve the contact between organic–inorganic interfaces, this addition effectively suppresses the crystallization of PEO. When Ta^5+^ replaces La^3+^, La vacancies will be generated, by creating a more uniform electrostatic environment, the activation energy required for Li⁺ hopping between different sites is substantially reduced.^[^ ^23^ ^]^ The characterization and simulation results indicate that cation vacancies optimize the dissociation of lithium salts and the hopping mechanism of lithium ions.^[^ ^26^ ^]^ To gain a deeper understanding of the temperature‐dependent interface dynamics and correlate it with the macroscopic electrochemical performance, we employ in situ conductive atomic force microscopy (c‐AFM). The optimized CPEs achieve a record ionic conductivity of 0.65 mS cm−1 at 60 °C, a Li⁺ transference number of 0.47, and exceptional cycling stability (93.39% capacity retention after 2000 cycles at 2C), offering a universal framework for advanced solid‐state battery design.

## 2 Results and Discussion

**Figure 1:**
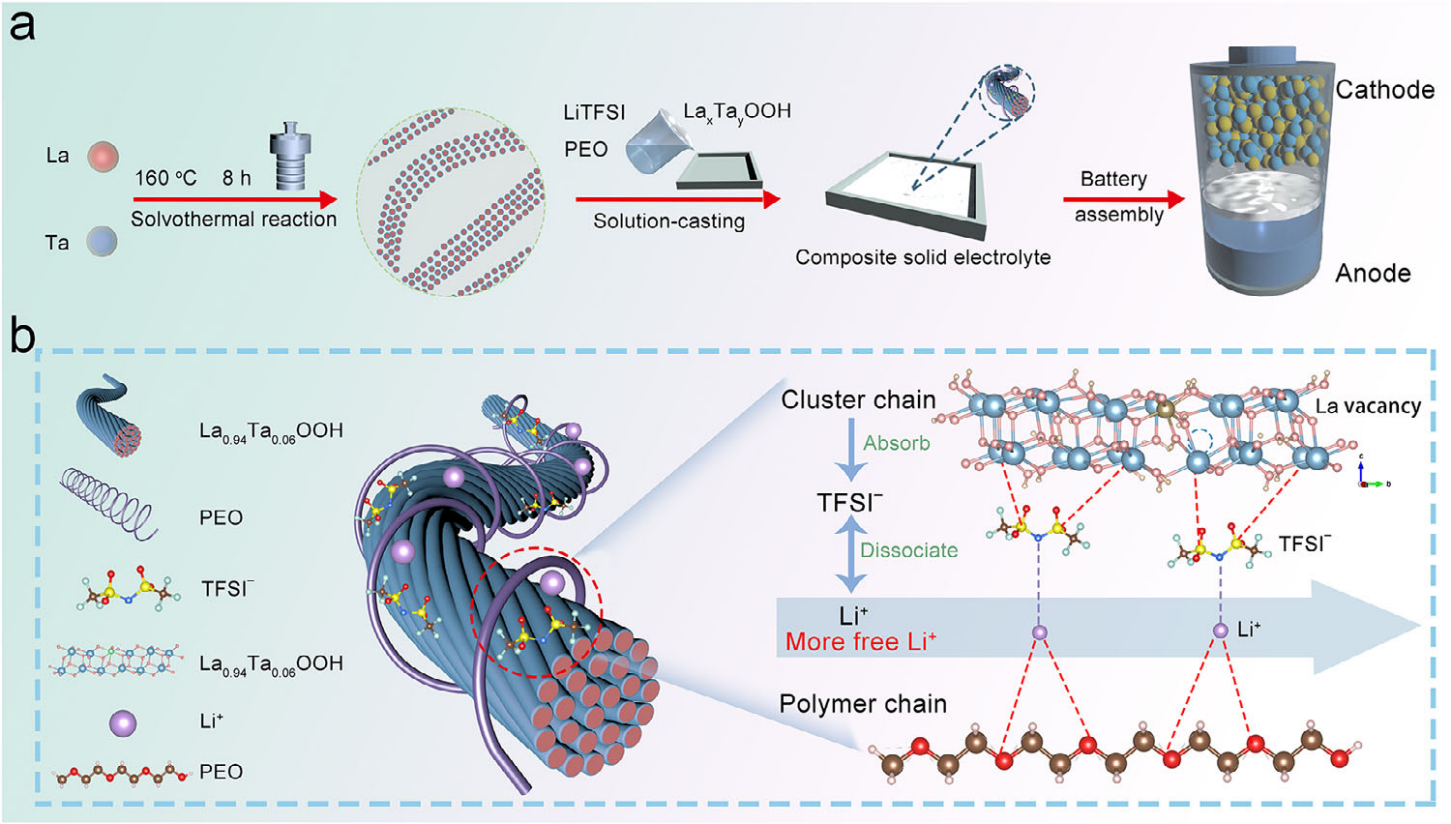
a) The schematic illustration for the preparation processes of the CPEs and assembled ASSLMBs using the CPEs. b) Various interactions of the electrolyte and mechanism for enhanced Li^+^ transport by La_0.94_Ta_0.06_OOH@PEO: LiTFSI.

**Figure** **1**a shows the synthesis pathway of La_x_Ta_y_OOH nanomaterials and the assembly process of all‐solid‐state batteries. Initially, an aqueous precursor system was established by co‐dispersing lanthanum and tantalum sources in deionized water. During nanocrystal growth, ethanol was introduced as a co‐solvent along with a dual‐ligand surfactant system comprising oleylamine and oleic acid. This dual‐ligand strategy synergistically modulates nucleation kinetics, where the long alkyl chains of oleic acid provide steric hindrance while the amino groups of oleylamine generate electrostatic repulsion.^[^ ^28^ ^]^ To optimize interfacial compatibility in organic–inorganic composites, we developed an innovative ternary solvent system (tetrahydrofuran‐dichloromethane‐acetonitrile). Dichloromethane is helpful to destroy the intermolecular force between oleamine molecules, and tetrahydrofuran is often employed to disperse oleic acid‐coated nanomaterials. Its cyclic structure helps to prevent the reaggregation of nanomaterial clusters. In addition, the solvent mixture can significantly dissolve PEO powder and lithium bis((trifluoromethyl)sulfonyl)azanide (LiTFSI). As demonstrated in Figure S1 (Supporting Information), this solvent system achieves highly uniform dispersion in PEO matrix while maintaining excellent optical transparency of LTPE film. The optimized electrolyte membranes have been successfully implemented in CR2032 coin cells and flexible pouch cell prototypes, with forthcoming electrochemical performance data expected to significantly advance solid‐state electrolyte industrialization.

The synthesized inorganic cluster chains demonstrate a propensity for directional self‐assembly, where surface‐grafted organic ligands prevent disordered aggregation via steric hindrance. Building upon the LaOOH matrix, we employed a high‐valent Ta⁵⁺ doping strategy, substituting La^3+^ lattice sites based on ionic radius matching principles to create La^3+^ vacancy defects. Theoretical calculations reveal that such cation vacancies significantly reduce Li^+^ migration activation energy through charge compensation mechanisms. Crucially, Ta^5+^ doping‐induced La^3+^ vacancies form three‐dimensionally interconnected networks, providing continuous diffusion pathways for Li⁺ transport.^[^ ^29^ ^]^ Research by Mai et al. reveals that unpaired electrons near cation vacancies transfer to TFSI^−^ anions, accelerating TFSI^−^ decomposition kinetics. The resulting inorganic‐rich solid electrolyte interface (SEI) film derived from TFSI^−^ demonstrates exceptional stability and enhanced kinetics for rapid, uniform lithium‐ion transport, thereby suppressing lithium dendrite growth.^[^ ^30^ ^]^ Meanwhile, positively charged lithium ions can form coordination complexes with oxygen atoms in PEO, which can enhance the mobility of lithium ions and promote ion conductivity, as shown in Figure 1b.^[^ ^31^ ^]^

**Figure 2:**
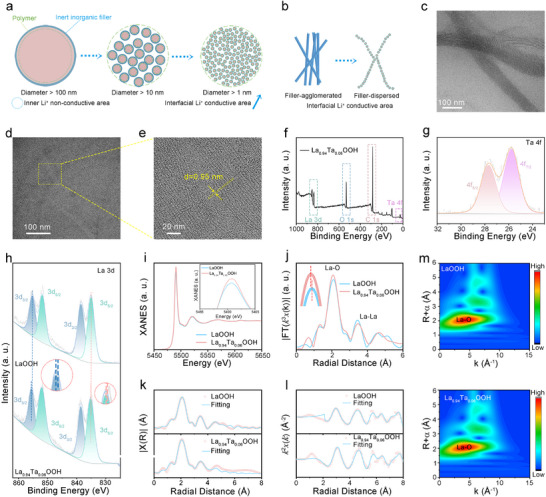
a) Schematic illustration of interface areas in CPEs with different sizes of fillers. b) Schematic illustration of efficient Li^+^ transport channels in fibrous filler and cluster chain filler. c) TEM image of LaOOH. d) TEM image of La_0.94_Ta_0.06_OOH. e) TEM image of one single inorganic cluster chain with a diameter of 0.95 nm. XPS spectra about the full spectrum f), Ta 4f g), and La 3d h) of La_0.94_Ta_0.06_OOH. i) The Fourier transforms (FT) curves of EXAFS for LaOOH and La_0.94_Ta_0.06_OOH. j) The La L‐edge FT curves of EXAFS of the samples. k,l) Fitting curves of the EXAFS of LaOOH and La_0.94_Ta_0.06_OOH in the R‐space and K‐space. m) WT‐EXAFS plot for LaOOH and La_0.94_Ta_0.06_OOH.

**Figure** **2**a schematically illustrates the size‐effect mechanism at organic–inorganic interfaces in CPEs. As the filler size is reduced to the submicron regime, the exponential increase in interfacial specific surface area dramatically decreases the volume fraction of Li⁺ transport dead zones, while the density of exposed active sites per unit volume improves by orders of magnitude. Consequently, this interfacial engineering strategy prioritizes organic–inorganic heterointerfaces as preferential pathways for rapid Li^+^ conduction through maximized effective conductive contact area. Figure 2b reveals the influence of microstructural topological optimization on the ion transport networks by comparing NWs with our designed highly dispersed cluster chain system: NW aggregation induced by van der Waals forces (left) causes ionic channel discontinuity, whereas surface charge‐modulated cluster chains (right). Under ideal dispersion conditions, inorganic cluster chains can achieve mon‐dispersity in mixed solvents and uniformly bind with organic polymer chains. Finally, the highly dispersed cluster chains formed a complete Li^+^ conductive network in the polymer matrix.^[^ ^32^ ^]^ In order to highlight that the evenly dispersed filler is more conducive to Li^+^ conduction, we obtained LaOOH with nanowire morphology by adjusting the proportion of oleic acid and oleamine and the reaction time, which is called NW. In this study, the incorporation of Ta^5+^ into the LaOOH lattice was aimed at introducing La vacancies. We conducted doping with 2%, 4%, 6%, and 8% of Ta^5+^. The actual chemical compositions of the as‐synthesized powders were quantified by Inductively Coupled Plasma Atomic Emission Spectrometry (ICP‐AES) and confirm the targeted doping levels (Table S1, Supporting Information). We conducted electron paramagnetic resonance tests on La_x_Ta_y_OOH (y = 0.02, 0.04, 0.06, and 0.08) with different doping levels (Figure S2, Supporting Information) and found that the vacancy concentration of La sites reached its peak at y = 0.06. From the perspective of charge balance, the substitution of Ta^5+^ promotes the formation of lanthanum vacancies to maintain electrical neutrality; These vacancies provide additional potential sites for lithium ions and reduce their migration barriers Therefore, the peak vacancy concentration measured at 6% is consistent with the observed maximum conductivity. (Figures S3 and S4, Supporting Information) At this point, compared to the total mass of PEO, LiTFSI, and La_0.94_Ta_0.06_OOH, the mass percentage of La_0.94_Ta_0.06_OOH is 10% by weight. We chose La_0.94_Ta_0.06_OOH as the final choice of electrolyte filler due to the non‐monotonic variation of comprehensive conductivity with Ta content and the peak vacancy density at 6%. Avoiding the structural distortion, crystal phase instability, or deterioration of cluster dispersion that may be caused by excessive doping.

The XRD spectra of NW, LaOOH, and La_0.94_Ta_0.06_OOH are consistent with the LaOOH crystal phase (Figure S5, Supporting Information), which is consistent with previous reports. The organic molecules on the surfaces of LaOOH and La_0.94_Ta_0.06_OOH were characterized and verified by Fourier transform infrared spectroscopy (Figure S6, Supporting Information). The absorption peaks appearing at ≈2851 and 2920 cm^−1^ indicate the stretching vibration of the C─H bond of the long carbon chain of oleylamine, while the CH_2_ bending vibration of the 1440 cm^−1^ peak. 1560 cm^−1^ is related to the stretching vibration of the C═C double bond in oleic acid. In order to achieve uniform composites, the morphology and dispersion characteristics of the chains were studied. The high‐resolution TEM images are shown in Figure 2c–e, where cluster chains tend to be arranged in bundles. Compared to the dispersion of LaOOH (Figure 2c), La_0.94_Ta_0.06_OOH (Figure 2d) has better dispersion and more atoms exposed on the surface per unit volume. The diameter of the synthesized inorganic carbon chain is ≈0.95 nm (Figure 2e). We conducted systematic high‐resolution TEM imaging across multiple sample regions. We systematically analyzed the dimensions of over 100 individual cluster chains that were randomly selected from nine TEM images obtained from three independent samples. Figure S7, Supporting Information). The measured average chain diameter is ≈0.95 nm, confirming high uniformity at the sub‐1 nm scale. From numerous TEM images, it can be seen that the lack of obvious aggregation of cluster chains proves its good dispersibility. In order to obtain the diameter size of the cluster chain more accurately, we conducted small‐angle X‐ray scattering (SAXS), as shown in Figure S8 (Supporting Information). There are two distinct peaks at q = 1.5 and q = 12.8 nm^−1^. According to TEM analysis, d = 2 π/q ≈ 0.5 nm at q = 12.8 is not the diameter of the cluster chain, but the size of the tightly arranged atomic core region inside the cluster.^[^ ^33^ ^]^ The 0.95 nm observed by TEM is the overall diameter of the atomic cluster nucleus and surface ligands. Calculate d = 2 π/q ≈ 4.2 nm at q = 1.5 is the spacing between each cluster chain. The presence of sub‐1 nm diameter clusters with high specific surface‐area helps to increase the migration channels of ions in solid electrolytes, thereby improving the overall ion conductivity. Small sized clusters can provide shorter ion transport paths, reduce resistance to ion migration, and improve electrolyte conductivity. SEM characterization was performed on LaOOH and La_0.94_Ta_0.06_OOH, and the corresponding mapping element distributions are shown in Figures S9 and S10 (Supporting Information). The prepared cluster chains have excellent dispersibility, which prepares for the subsequent preparation of homogeneous solid electrolyte membranes. Notably, NW samples without optimized ligand ratios (Figure S11, Supporting Information) maintain nanowire bundle aggregation due to insufficient electrostatic stabilization.

X‐ray photoelectron spectroscopy (XPS) depth profiling provides compelling evidence for the precise regulation of surface chemical states in the synthesized materials. As demonstrated in Figure S12 (Supporting Information), the survey and high‐resolution spectra (La 3d, O 1s, C 1s) of LaOOH closely correspond to the characteristic features of La^3+^ oxyhydroxides reported in literature. The survey XPS spectrum of La_0.94_Ta_0.06_OOH (Figure 2f) unambiguously shows the characteristic Ta 4f peaks, verifying successful Ta doping. Deconvolution of Ta 4f spectrum (Figure 2g) reveals: The 1.9 eV splitting between Ta 4f_7/2_ (25.78 eV) and Ta 4f_5/2_ (27.68 eV), along with binding energy shifts,^[^ ^34^ ^]^ confirms the existence of Ta^+5^ and forming [TaO_6_] octahedral coordination with lattice oxygen.^[^ ^35^ ^]^ This electronic configuration is expected to substantially enhance Li^+^ mobility via locally built‐in electric fields. The substitution of higher‐valent Ta^5+^ at La^3+^ sites necessitated charge compensation, primarily achieved through the formation of lanthanum vacancies (V_La_). Furthermore, the higher electronegativity of Ta compared to La promotes the formation of distinct La─O─Ta linkages. These structural modifications induce significant electronic redistribution. Specifically, the absence of La atoms connected via shared oxygen ligands, perturbs the electronic environment of adjacent La sites, effectively reducing the electron density around these remaining La cations. This electronic redistribution shifts the La 3d binding energies to higher values, as shown in the La 3d XPS spectra of LaOOH and La_0.94_Ta_0.06_OOH (Figure 2h), specifically from 855.28 to 855.58 eV (3d_3/2_) and 835.08 to 835.18 eV (3d_5/2_).

To further elucidate the atomic‐scale coordination environment, X‐ray absorption near‐edge structure (XANES) and extended X‐ray absorption fine structure (EXAFS) spectroscopy were conducted. As shown in Figure 2i, the La L‐edge XANES spectrum of La_0.94_Ta_0.06_OOH exhibits higher peak intensity than LaOOH, indicating an increased oxidation state of La, consistent with the XPS results.^[^ ^36^ ^]^ The EXAFS spectra (Figure 2j) reveal distinct changes in the nearest La─O coordination shell and the next‐nearest La–La shell: La_0.94_Ta_0.06_OOH shows slightly enhanced La‐O peak intensity but reduced La–La peak intensity relative to LaOOH.^[^ ^37^ ^]^ Combined with EXAFS fitting results (Figure 2k,l; Table S2, Supporting Information), the shortened La─O bond length and intensified peak amplitude in La_0.94_Ta_0.06_OOH demonstrate the substantial presence of V_La_.^[^ ^38^ ^]^ This conclusion is further corroborated by wavelet transform (WT) analysis (Figure 2m). The maximum WT signal at ≈ 4.10 Å^−1^ in K‐space corresponds to the La─O path, while the La─O bond lengths derived from R‐space fitting are 2.11 Å for LaOOH and 2.08 Å for La_0.94_Ta_0.06_OOH.^[^ ^39^ ^]^ These shortened bonds and altered local atomic structures (evidenced by modified oscillations in the 4–8 Å^−1^ region) confirm that Ta doping induces V_La_ and modifies La─O bonding.

**Figure 3:**
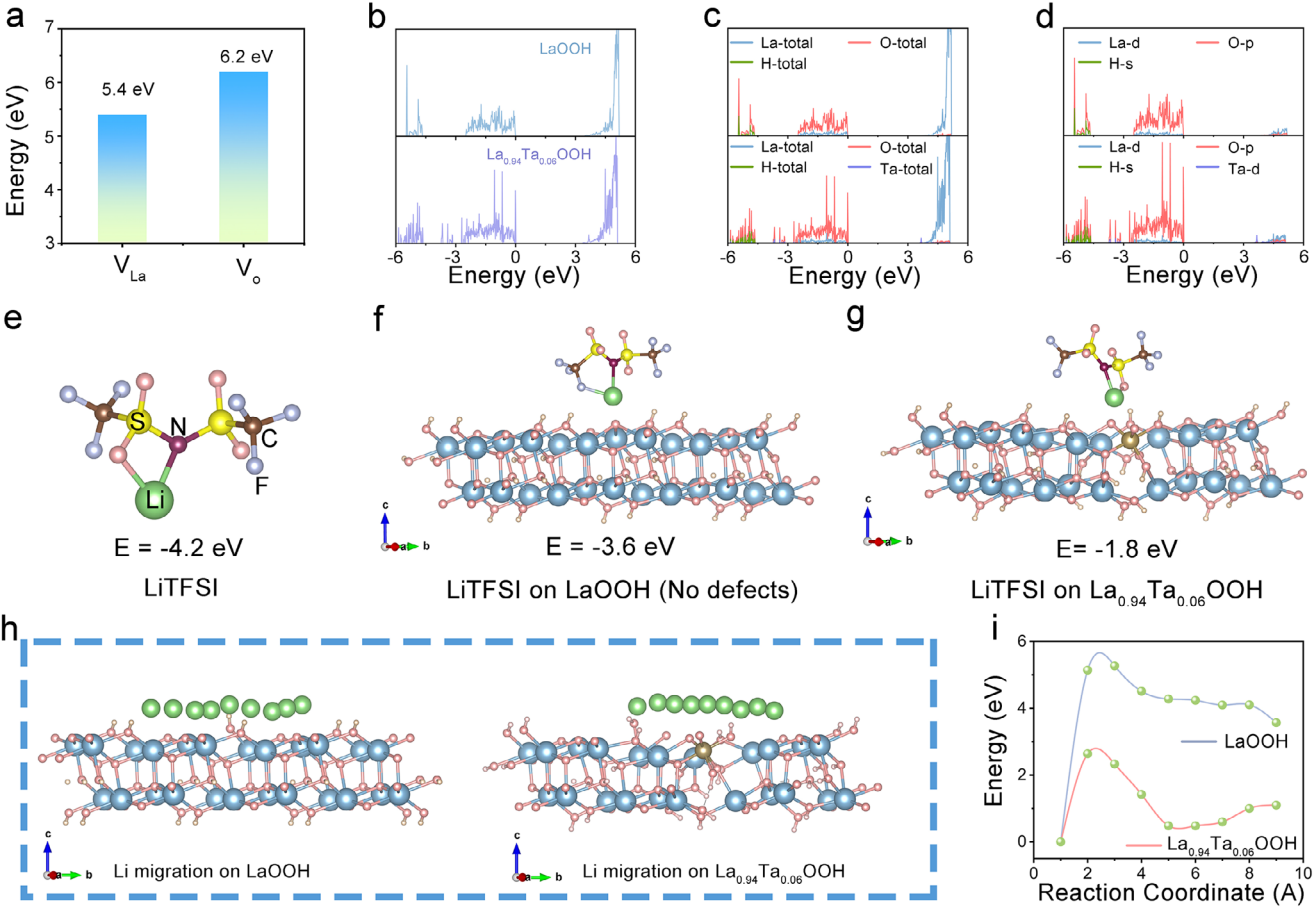
a) Calculated enthalpies of null generation for lanthanum and oxygen vacancies. b–d) Density of states spectra of LaOOH and La_0.94_Ta_0.06_OOH structures. The binding energy of Li^+^ in e) free LiTFSI molecule, and in f) LiTFSI on the surface of LaOOH, and g) LiTSFI on La_0.94_Ta_0.06_OOH. h) Migration mechanism of Li^+^ in LaOOH, and La_0.94_Ta_0.06_OOH. Based on DFT i) Migration energy barrier f Li^+^ in LaOOH and La_0.94_Ta_0.06_OOH.

To elucidate the role of V_La_, the density functional theory (DFT) calculations were performed on both defect‐free LaOOH and defect‐rich La_0.94_Ta_0.06_OOH models. The calculated formation enthalpies reveal that the generation of VLa is energetically more favorable than that of an oxygen vacancy (V_o_), as illustrated in **Figure** **3**a. Figure 3b presents the density of states (DOS) for LaOOH and La_0.94_Ta_0.06_OOH. It is worth noting that the bandgap of La_0.94_Ta_0.06_OOH is narrower than that of LaOOH.^[^ ^40^ ^]^ This reduction is primarily attributed to the lower energy position of the Ta^5+^ 5d orbitals relative to the La^3+^ 5d orbitals upon doping, which results in a downshift of the conduction band minimum (CBM). Analysis of the projected DOS in Figure 3c,d indicates that states in the conduction band region (≈3–5 eV) are dominated by La d‐orbitals, while those near the valence band maximum (≈−3–0 eV) originate predominantly from O p‐orbitals.^[^ ^41^ ^]^ The observed electronic restructuring suggests potential implications for enhancing ionic transport, likely mediated through improved charge compensation or modified defect chemistry.^[^ ^42^ ^]^

The metal V_La_ facilitates ion transport by introducing defect sites within the solid electrolyte, thereby creating more efficient conduction pathways. These vacancies serve as preferential hopping sites for ion hopping. In order to investigate the interface effect between cluster chains and lithium salts, we conducted DFT calculations to analyze the binding energy between cluster chain surfaces and lithium salts in solid‐state electrolyte thin films. Figure 3e–g presents the binding energies between Li⁺ and TFSI^−^ ions. Reduced binding energies signify weakened ion‐pair interactions, facilitating enhanced ion dissociation. This trend indicates a strengthening of Lewis acid‐base interactions.^[^ ^43^, ^44^ ^]^ Notably, cation vacancies in La_0.94_Ta_0.06_OOH mediate a 50% reduction in the Li⁺ dissociation energy compared to the LaOOH, significantly enhancing ionic mobility. Further exploration was conducted on the migration path and corresponding migration energy barrier of lithium ions on the surface of clusters (Figure 3h,i). In solid‐state ion diffusion, Li⁺ migrates via a vacancy‐mediated mechanism, wherein a Li⁺ can only hop into an adjacent vacant site. If the target site is already occupied, the Li⁺ must expend additional energy to displace the resident ion, a process that constitutes the major component of the activation energy barrier. The cation vacancies are essentially pre‐existing vacant sites within the filler lattice that are available for Li⁺ occupation. The cation vacancies within the cluster chains serve as hopping sites for Li⁺, thereby reducing the activation energy for ion migration. When these clusters interconnect, they form a 3D percolating network that establishes continuous low‐energy pathways for lithium transport. Introducing the tantalum element helps to reduce atomic packing density, thereby reducing spatial effects and electrostatic repulsion during Li migration, and lowering the migration energy barrier. As shown in Figure 3i, the maximum energy barrier has decreased by ≈80%, significantly improving the Li migration mechanics.

**Figure 4:**
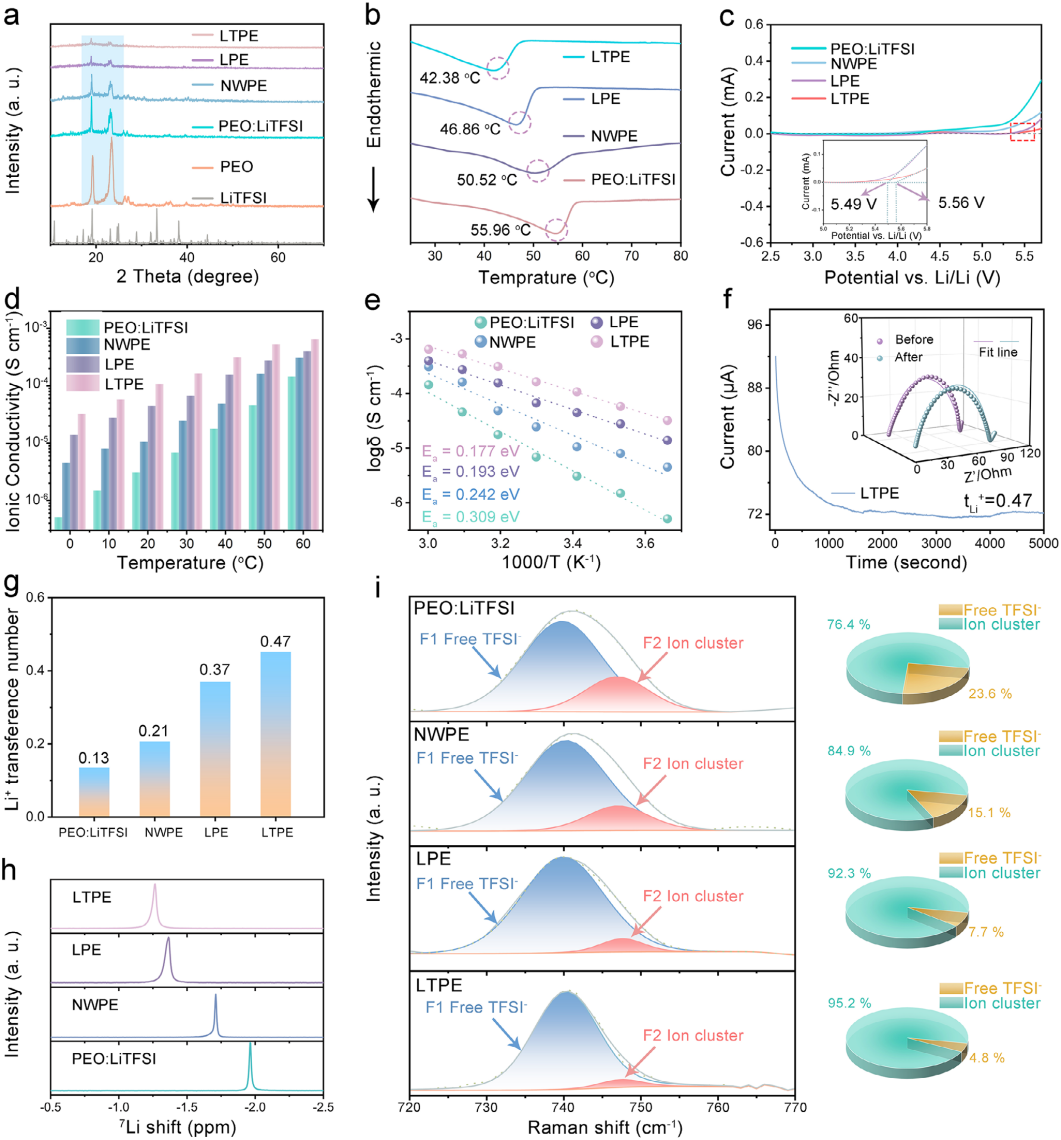
Electrochemical performance of electrolyte membrane (LiTFSI, PEO, PEO: LiTFSI, NWPE, LPE, and LTPE) a) XRD patterns. b) DSC profiles. c) LSV curves. d) Ionic conductivity at different temperatures. e) Arrhenius plots at different temperatures. f) *I–T* curves of Li||LTPE||Li cells at 10 mV. The inserted graph is the EIS curves before and after polarization. g) Li^+^ Transference Number h) ^7^Li NMR spectra. i) Raman spectra and the proportion of Free TFSI^−^ and Ion cluster.

In the polymer composite electrolyte system, we use La_0.94_Ta_0.06_OOH as the best filler, and the filler quality fractions of 5%, 10%, 15%, and 20% were investigated. (Figure S13, Supporting Information) The results showed that 15 wt.% was the optimal proportion, exhibiting the best electrochemical performance (Figure S14, Supporting Information) Analyzing the solvent residue situation of the electrolyte membrane, we conducted thermogravimetric analysis (TGA) on the optimized solid electrolyte membrane. The TGA curve (Figure S15, Supporting Information) shows no significant weight loss below 200 °C, which is much higher than the typical boiling points of solvents used in electrolyte processing (tetrahydrofuran, dichloromethane, and acetonitrile). This confirms the complete removal of solvents during our drying process. Further observation of the microstructure of electrolyte membranes prepared from LaOOH and La_0.94_Ta_0.06_OOH samples through SEM characterization. The elemental distribution of La, Ta, O, and C under microscopic conditions was analyzed using EDS spectra, and the results are shown in Figures S16 and S17 (Supporting Information). The Ross‐section scanning mapping image shows that the various elements in LTPE are also evenly distributed.(Figure S18, Supporting Information) As evidenced by the elemental mapping of the membrane's surface and cross‐section, La_0.94_Ta_0.06_OOH is uniformly distributed within the electrolyte membrane. The SEM image of the thickness of the CPEs is shown in Figure S19 (Supporting Information), and the thickness of the film is ≈180 µm. Dissolve LiTFSI, PEO, and NW/LaOOH/La_0.94_Ta_0.06_OOH in a mixed solvent system of tetrahydrofuran, dichloromethane, and acetonitrile to form a PEO‐based solid electrolyte. The pure polymer solid electrolyte is labeled as PEO: LiTFSI, and the prepared electrolyte doped with NW, LAOOH and La_0.94_Ta_0.06_OOH is referred to as NWPE, LPE, and LTPE, respectively. XRD analysis was employed to elucidate the filler‐mediated suppression of polymer crystallization within the PEO‐based matrix (**Figure** **4**a). Comparative study of diffraction patterns for PEO powder, PEO: LiTFSI, and composite systems (NWPE/LPE/LTPE) reveals gradient attenuation in relative intensity of characteristic peaks at 19°and 23°. The LTPE exhibits the most pronounced crystallinity reduction, primarily attributed to strong Lewis acid‐base interactions between La_0.94_Ta_0.06_OOH surface sites and PEO ether oxygen groups. Differential scanning calorimetry (DSC) shows (Figure 4b) the melting temperature of LTPE (42.38 °C) decreases by 23.4% compared to pristine PEO (55.96 °C), indicating filler‐polymer interfacial interactions effectively suppress chain recrystallization.^[^ ^45^ ^]^ In situ tensile testing (Figure S20, Supporting Information) demonstrates that LTPE maintains structural integrity at 130% strain with fracture elongation. This result indicates that La_0.94_Ta_0.06_OOH can significantly reduce the crystallinity of PEO‐based solid electrolytes, which helps to improve the overall thermal stability of the material. From the LSV curve in Figure 4c, it can be seen that compared to PEO: LiTFSI, LTPE exhibits a voltage window of 5.65 V. A wide voltage window can reduce the chance of side reactions during battery charging and discharging, help improve the thermal stability of electrolytes, and reduce the risk of thermal runaway of batteries under high‐temperature or overcharge conditions, which is crucial for electric vehicles and energy storage systems. Ion transport properties, as critical determinants of rate capability and cycling stability in all‐solid‐state lithium metal batteries, necessitate in‐depth mechanistic investigation. Through symmetric blocking cell measurements (Figure S21, Supporting Information) coupled with equivalent circuit modeling (Figure S22, Supporting Information), we systematically elucidated the topological regulation of ionic conduction in PEO‐based electrolytes.

As shown in Figure 4d, LTPE exhibits exponential enhancement in ionic conductivity across 0–60 °C: reaching 3.2 × 10^−5^ S cm^−1^ at 0 °C (62.8‐fold improvement vs PEO: LiTFSI) and 6.5 × 10^−4^ S cm^−1^ at 6 °C (4.5‐fold improvement PEO: LiTFSI). To better evaluate the performance of the filler developed in this work, a comparative analysis of its ionic conductivity with that reported in other studies was conducted. (Table S3, Supporting Information) Arrhenius analysis (Figure 4e) confirms significantly reduced activation energy of 0.177 eV for LTPE (42.72% decrease), demonstrating effective interfacial engineering in lowering Li⁺ migration barriers through Ta doping. To unravel Li⁺ transport dynamics, chronopotentiometry EIS combined measurements (Figure 4f; Figure S23, Supporting Information). As shown in Figure 4g, the lithium ion migration numbers of PEO: LiTFSI, NWPE, LPE, and LTPE are 0.13, 0.21, 0.37, and 0.47, respectively. As a filler for PEO‐based solid electrolytes, La_0.94_Ta_0.06_OOH is significantly superior to the LAOOH. From this, it can be seen that thanks to the presence of V_La_ as abundant Lewis acid sites, a large number of lithium ions can move freely through the adsorption of TFSI^−^. Regrettably, the lithium‐ion transference number of 0.47 remains relatively low compared to state‐of‐the‐art studies. To enhance the performance of next‐generation polymer electrolytes, we plan to design novel polymer frameworks by incorporating positively charged groups into the chain—through grafting or cross‐linking—to effectively restrict anion migration and thereby significantly increase the lithium‐ion transference number.

To elucidate the regulatory mechanism of La_0.94_Ta_0.06_OOH fillers on Li⁺ transport dynamics, we employed high‐resolution 7Li nuclear magnetic resonance (7Li NMR) to probe the evolution of lithium ion local chemical environments (Figure 4h).^[^ ^46^ ^]^ Compared to the baseline PEO system (δ = −1.96 ppm), LTPE exhibits a downfield shift of the 7Li signal to δ = −1 ppm. This de‐shielding effect corresponds to a significant reduction in Li⁺‐ether oxygen coordination binding energy. According to the Knight shift theory, this chemical environment modification confirms weakened Li⁺‐polymer coordination with increased free volume fraction. In order to fully explore the additional interface effect in organic–inorganic composites, we carefully studied the LiTFSI dissociation performance in solid electrolyte by Raman spectroscopy. Therefore, it can be expected that the interfacial transport kinetics will improve. As evidenced by Figure 4i, the left peak corresponds to free TFSI^−^ ions, and the right peak represents undivided LiTFSI.^[^ ^47^ ^]^ The increase of free TFSI^−^ ion content in LTPE can be attributed to Lewis acid‐base interaction. The illustration on the right side of Figure 4i shows the proportion of free TFSI^−^ and undivided TFSI^−^ of various electrolytes. Specifically, the high dissociation of LiTFSI indicates that the addition of La_0.94_Ta_0.06_OOH forms a uniform and continuous organic–inorganic interface, which results in the highest degree of LiTFSI dissociation. The increase of lithium salt dissociation degree provides an additional way for lithium ion transport. the morphology and dispersion characteristics of the chains were studied. We further conducted infrared testing on the electrolyte. The C─O─C stretching vibration peak of pure PEO electrolyte is observed at 1056.8 cm^−1^ (characteristic of ether‐oxygen chains). In the LTPE composite electrolyte (with La_0.94_Ta_0.06_OOH), this peak exhibits a significant low‐frequency shift to 1053.4 cm^−1^ (Figure S24, Supporting Information).^[^ ^48^ ^]^ This shift indicates electron density redistribution between La_0.94_Ta_0.06_OOH surface sites and PEO ether‐oxygen atoms (‐O‐), reducing C─O bond strength and directly confirming interfacial coordination bonding. According to the Lewis acid‐base coordination principle, the small radius and high charge density of Lewis acids such as La^3+^ and Ta^5+^ can preferentially form strong electrostatic and directional coordination bonds with the ether oxygen in PEO, thereby competitively occupying the ether oxygen sites originally available for coordinating with Li^+^. When high‐valence cations occupy the ether oxygen atoms, they deplete the coordination environment of Li^+^, resulting in reduced electron density from its solvation shell and a weakened shielding effect. This leads to a transition of ^7^Li NMR spectra toward low field.^[^ ^49^ ^]^ The weakening of coordination strength implies that the solvation shell of Li^+^ is more likely to become loose or partially detached, a process known as de‐solvation, which reduces the migration energy barrier for Li^+^ in the electrolyte.^[^ ^50^ ^]^ Therefore, it can be expected that the interfacial transport kinetics will improve, which is consistent with the revious calculation results.

**Figure 5:**
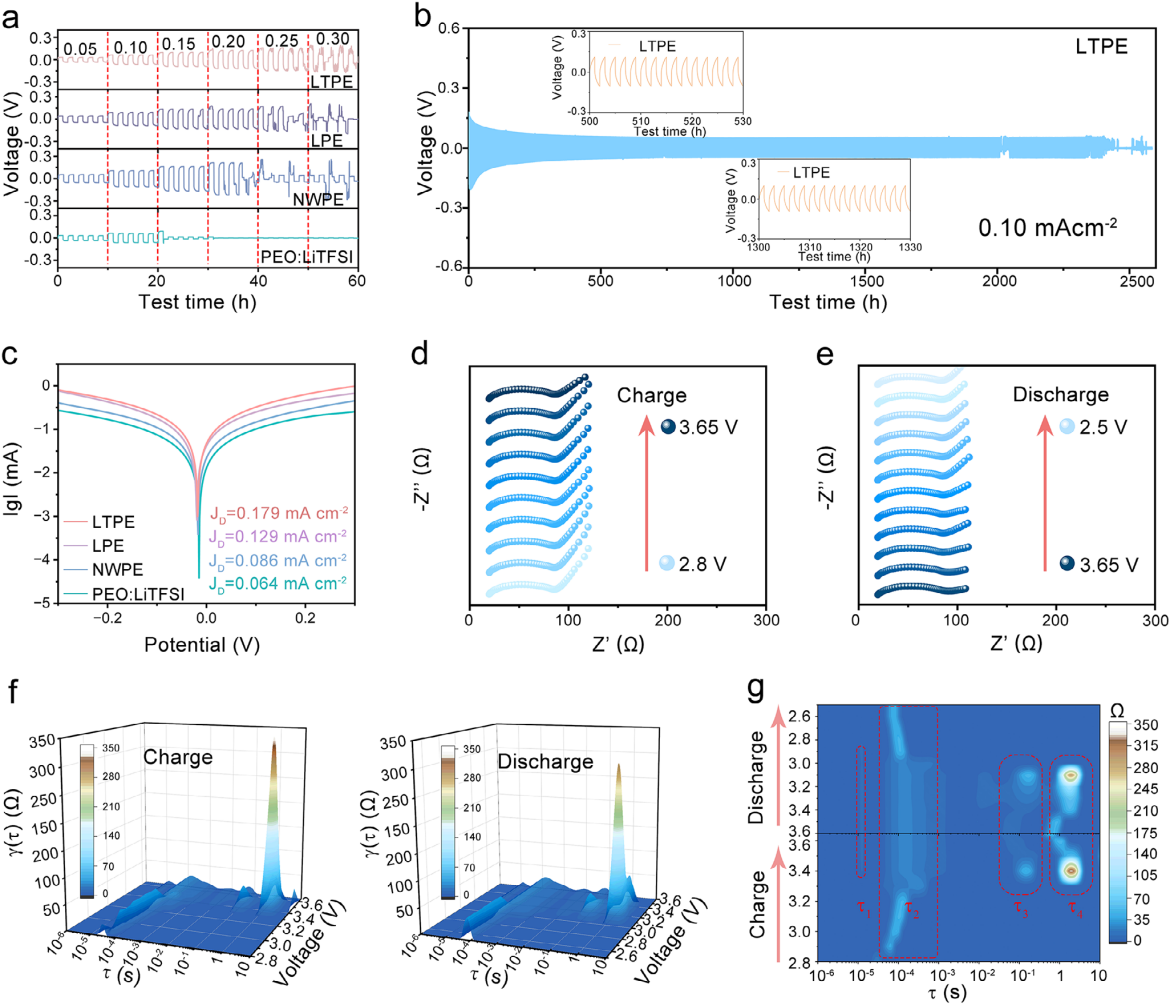
The performance of Li||electrolytes||Li batteries. a) Critical current density from 0.05 to 0.3 mA cm^−2^; b) cycling performance at 0.1 mA cm^−2^; c) Tafel plots. EIS of LFP||electrolytes||Li batteries with LTPE at different voltage charge d) and discharge e). f) The DRT transform of the 3D spectrum of EIS during the charge and discharge process. g) The DRT transformation of EIS spectra.

This work systematically investigates the electrochemical stability of ASSLMBs under varying current densities. Through Li–Li symmetric cell cycling tests (current density: 0.05–0.30 mA cm^−2^), it was demonstrated that lithium batteries incorporating LTPE maintain stable operation under increasing current densities. Notably, when reaching critical current densities, cells employing LPE, NWPE, and PEO: LiTFSI experienced short‐circuit failures (**Figure** **5**a), providing compelling evidence that homogeneous and continuous organic–inorganic interfacial structures are crucial for maintaining electrochemical stability at elevated current densities. However, when the current density is increased to 0.3 mA cm^−^^2^, it induces a substantial rise in local stress during lithium deposition. The LNPE fails to effectively withstand this elevated stress, leading to the formation of microcracks at the interface. These microdefects provide favorable conditions for non‐uniform lithium deposition and dendrite growth, ultimately resulting in dendrite penetration through the separator and the occurrence of a soft short circuit. Further lithium deposition/stripping tests in symmetric cells (60 min cycling per current density) revealed exceptional long‐term stability exceeding 2000 h at 60 °C and 0.1 mA cm^−2^ for LTPE (Figure 5b), with stable polarization voltages maintained after 500 and 1300 cycles (inset), conclusively demonstrating the superior lithium metal compatibility of cluster‐chain modified PEO‐based electrolytes. The electrochemical performance of the LTPE filler was systematically compared with representative fillers reported in the literature. This comparative assessment focused on three key metrics: ionic conductivity, lithium‐ion transference number, and long‐term cycling stability in lithium symmetric cells. The corresponding data are compiled in Table S4 (Supporting Information).

To elucidate the impact of cluster‐chain electrolytes on lithium‐ion transport kinetics, galvanostatic intermittent titration technique (GITT) measurements were conducted. Results indicated that the LTPE system exhibited significantly higher lithium‐ion diffusion coefficients (D') compared to control groups (Figure S25, Supporting Information). This enhanced ionic diffusivity effectively suppressed voltage polarization during cycling, resulting in the lowest and most stable polarization characteristics for the LTPE system (Figure 5c; Figure S26, Supporting Information).^[^ ^51^ ^]^ Tafel analysis derived from cyclic voltammetry (CV) measurements further confirmed that La_0.94_Ta_0.06_OOH‐modified PEO electrolytes increased the exchange current density from 0.0.64 to 0.179 mA cm^−2^, indicating accelerated ion transport kinetics at the LTPE interface. Additionally, CV tests on LiFePO_4_/Li full cells at various scan rates (0.1–1.0 mV s^−1^, voltage window: 2.8–4.0 V) revealed well‐defined Fe^2+^/Fe^3+^ redox couple peaks at 3.32 and 3.53 V for the LiFePO_4_/Li system (Figure S27, Supporting Information), demonstrating excellent electrochemical reversibility.

To gain a more in‐depth understanding of the evolution of electrical properties during charge–discharge processes, in situ charge–discharge resistance tests were conducted (Figure 5d,e).^[^ ^52^ ^]^ These measurements were specifically designed to dynamically monitor the evolution of the electrolyte's resistance under realistic operating conditions. The typical EIS spectra comprise three main regions: the internal resistance (represented by the high‐frequency intercept on the real axis), the interfacial impedance (represented by the semicircle in the high‐to‐medium frequency range), and the diffusion impedance (Warburg impedance). The basic processes in batteries mainly include conduction‐based processes, charge transfer‐based processes, physical contact, and diffusion processes, which are also unique in their specific systems. As shown in Figure 5f,g, the DRT (Distribution of Relaxation Times) analysis reveals four main peaks.^[^ ^53^ ^]^ The peak at a time constant of τ1 ≈ 10^−5^ s is attributed to Li⁺ conduction in the polymer electrolyte. The peak at τ2 ≈ 10^−4^ s is associated with the dynamic evolution of the SEI film, reflecting the migration of Li⁺ and reconstruction of the interfacial layer.^[^ ^54^ ^]^ The peak at τ3 ≈ 10^−1^ s corresponds to the charge transfer impedance at the LiFePO_4_ (LFP) cathode/electrolyte interface, while the peak at τ4 is ascribed to the solid‐state ionic diffusion impedance within the LFP active material. In the DRT contour plot, strong peaks are denoted by brown regions, and weak peaks by blue regions. In situ impedance and DRT analysis spectra of LFP||LPE||Li, LFP||NWPE||Li, and LFP||PEO:LiTFSI||Li were shown in Figures S28–S30 (Supporting Information). Compared to the LTPE battery, the PEO battery exhibited significantly higher interfacial impedance, which can be attributed to interfacial side reactions at the electrode‐electrolyte interface. In contrast, the stable interfacial modification in the LTPE battery facilitated uniform ion transport, enabling reversible lithium plating/stripping and ion intercalation/deintercalation. This achievement lays a solid foundation for enhanced cycling stability.

In order to deeply analyze the mechanism of temperature regulation on ion transport dynamics of solid electrolyte, in situ c‐AFM was used to analyze the voltage response of LTPE electrolyte in the temperature range (30–50 °C). In this experimental study, a half‐cell system was meticulously constructed, consisting of an electrolyte, a lithium‐metal anode, and a conductive AFM probe. By applying a precisely defined voltage, the migration of lithium ions in the electrolyte is achieved. At 30 °C, a substantial amount of chain‐like crystalline PEO was observable within the electrolyte. When the temperature was incrementally raised to 50 °C, the electrolyte exhibited no discernible changes. Nevertheless, upon reaching 60 °C, a remarkable transformation occurred: the chain‐like PEO underwent a transition into an amorphous state (Figure S31a, Supporting Information). Figure S31b (Supporting Information) displays the corresponding 3D representation, which provides an intuitive visualization of the PEO‐based electrolyte's phase transition. This 3D diagram clearly reveals that at 60 °C, the PEO matrix transitions from a chain‐like crystalline structure into a viscous fluid, indicating a crystalline‐to‐amorphous phase transition. This transition has significant implications for the ion‐transport properties of the electrolyte, as the change in the PEO state can potentially alter the pathways and ease of lithium‐ion migration. For instance, the amorphous state may provide more flexible and disordered channels for Li⁺ movement, which could enhance the ionic conductivity of the electrolyte system, an aspect that warrants further in‐depth investigation.

**Figure 6:**
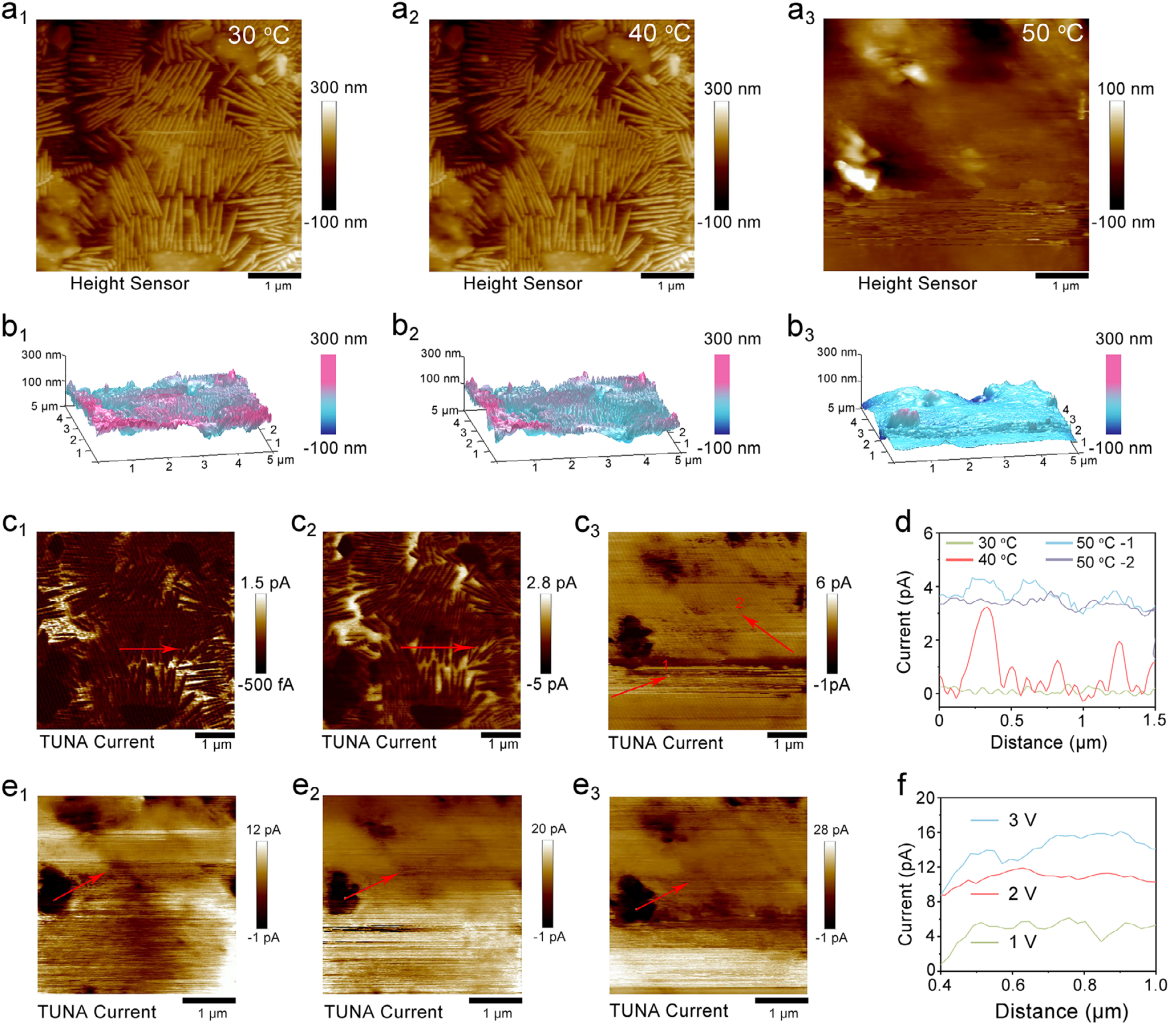
In situ c‐AFM characterization of LTPE electrolyte at a_1_) 30 °C, a_2_) 40 °C, and a_3_) 50 °C. b_1_–_3_) Corresponding 3D mode diagram. AFM morphology of c‐AFM current under c_1_) 30 °C, c_2_) 40 °C, and c_3_) 50 °C. d) c‐AFM current curve at grain boundaries in Figure 6c_1–3_. c‐AFM current under e1) 1 V, e2) 2 V and e3) 3 V at 50 °C. f) c‐AFM current curve at grain boundaries in Figure 6 e_1–3_.

When La_0.94_Ta_0.06_OOH was incorporated into the system, substantial crystallization was still evident at 30 and 40 °C. A distinct transition from a solid to a viscous fluid state, however, was observed upon heating to 50 °C (**Figure** **6**a,b). Notably, current could be sporadically detected even in the absence of an applied voltage. The main factor causing this phenomenon is inherent bias in any electronic system, and the temperature will never be precisely at absolute zero. At non‐zero temperatures, electrons have a certain amount of thermal energy, which may lead to the generation and movement of authigenic load carriers, albeit to a small extent. Concurrently, a contact potential difference between the c‐AFM tip and the electrolyte surface can establish an intrinsic electric field, which facilitates the directional drift of charge carriers without any external polarization.

We acquired the current images of the LTPE at various temperatures without applying an external voltage (Figure 6c). The variation in current can be clearly discerned from the image scale. The brightness of the corresponding image area is augmented. Quantitatively, the average current at 50 °C was measured to be 3.8 pA, which is markedly higher than the values recorded at 30 and 40 °C. This data strongly suggests that temperature exerts a significant influence on the migration of both ions within the LTPE (Figure 6d). At 30 °C stable temperature state, apply voltage levels of 1, 2, and 3 V to the LTPE in sequence. By using c‐AFM equipment, real‐time acquisition and recording of corresponding c‐AFM morphology images and corresponding current images are shown in Figure S32a,b (Supporting Information). From the experimental results, it can be observed that as the applied voltage gradually increases from 1 to 3 V, a detailed analysis of the current data in Figure S32c (Supporting Information) clearly shows a corresponding increasing trend in current, revealing an exponential current increase from 3.88 pA at 1V to 8.14 pA at 3V. At 50 °C (Figure 6e; Figure S33, Supporting Information), the surface roughness decreases significantly, confirming a partial crystalline‐to‐amorphous transition. As shown in Figure 6f, the current densities at 50 °C under 1–3V biases show remarkable enhancement compared to 30 °C, with the 3V bias current increasing to more than 12 pA (50 °C). This crystalline‐to‐amorphous transition elevates ionic conductivity to 2.1 × 10^−^^3^ S cm^−1^ at 50 °C, representing a 2.4‐fold increase over 30 °C (8.7 × 10^−^^4^ S cm^−1^). Thus, a 3D continuous ion transport channel is constructed, and the optimized multi‐scale ion transport dynamics is finally realized. In contrast, the pure PEO film shows only weak current signals, providing further evidence of the role of the cluster chains in conductivity. (Figure S34, Supporting Information) These results not only demonstrate how the cluster chains provide interconnected pathways for Li⁺ but also offer direct 2D visualization of the conductive network.

**Figure 7:**
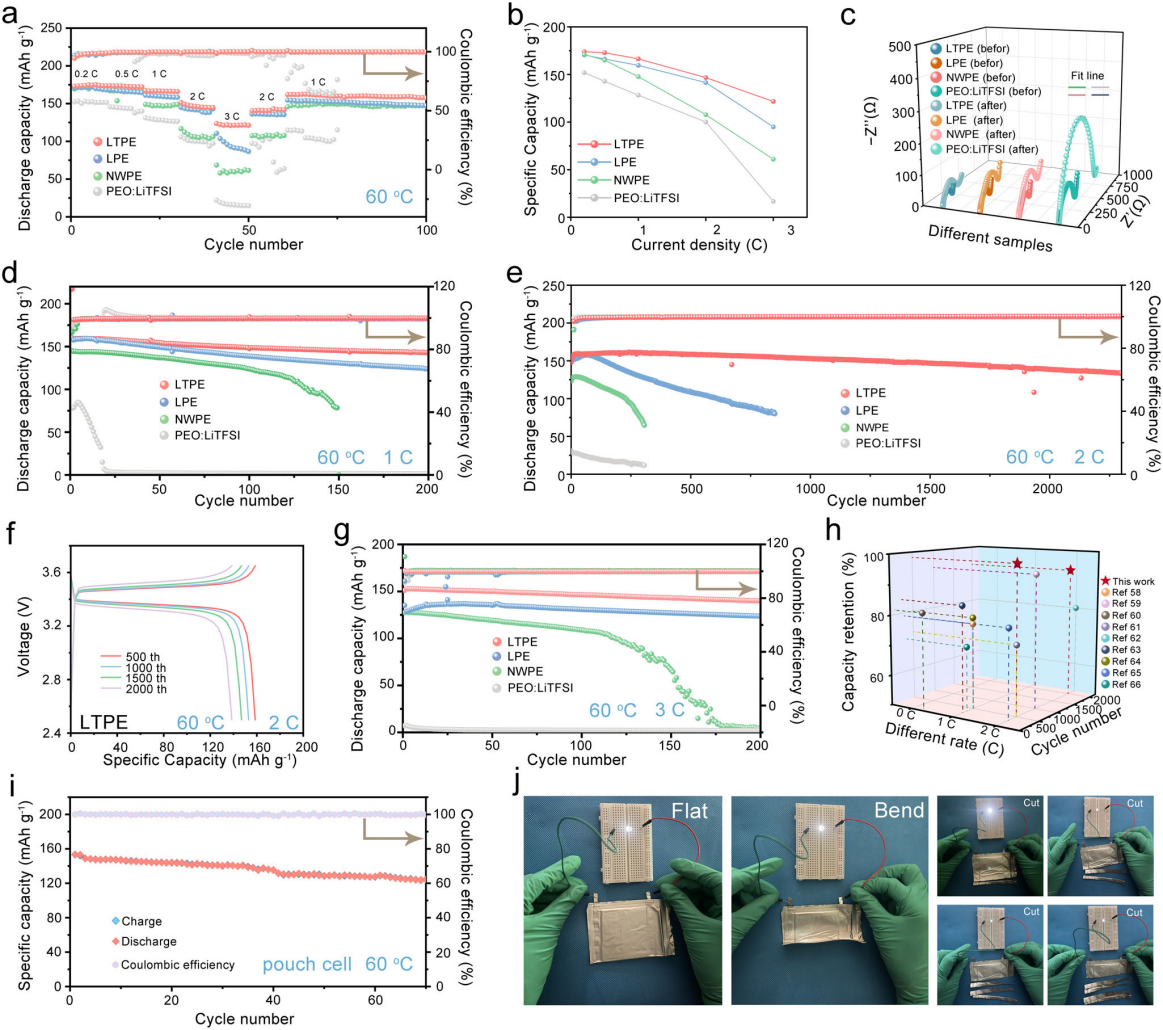
Rate capability (0.2–3C) with LFP||PEO: LiTFSI||Li, LFP||NWPE||Li, LFP||LPE||Li, and LFP||LTPE||Li operated 60 °C a) and the corresponding capacity b). c) The EIS curves of LFP||Li solid‐state full cells before and after 100 cycles at 1 C. d,e) Long‐life cycling performance of PEO: LiTFSI, NWPE, LPE, and LTPE electrolyte‐based LFP||Li batteries under the current density of 1C and 2C. f) The capacity voltage curve corresponding to LFP||LTPE||Li at 2C after different cycles. g) Long‐life cycling performance of PEO: LiTFSI, NWPE, LPE, and LTPE electrolyte‐based LFP||Li batteries under the current density of 3C. h) Electrochemical performance comparison with previous works. i) Cycling stability of pouch cells. j) Photographs of the pouch cells lighting LEDs original after folded and cut. (1C = 170 mAh g^−1^).

To systematically evaluate the practical performance of CPEs, we constructed LFP||Li cells with PEO:LiTFSI, NWPE, LPE, and LTPE electrolytes for comprehensive electrochemical characterization. As shown in **Figure** **7**a, the rate capability tests demonstrate that the LTPE‐based cell exhibits exceptional kinetic properties within 0.2–3C current densities, delivering stable discharge capacities of 173.9 (0.2C), 172.8 (0.5C), 166.4 (1C), 146.7 (2C), and 121.7 (3C). Remarkably, it retains 153.3 mAh g^−1^ when reverting to 1C. In contrast, the PEO: LiTFSI system shows inferior performance with an initial capacity of 151.9 mAh g^−1^ at 0.2C and rapid decay to 16.7 mAh g^−1^ at 3C (Figure 7b). EIS analysis using equivalent circuit modeling (Figure 7c, equivalent circuit in Figure S22, Supporting Information) quantitatively reveals interfacial impedance evolution. The LTPE‐based cell demonstrates the lowest ohmic resistance and charge transfer resistance, significantly outperforming PEO:LiTFSI, NWPE, and LPE systems. Notably, the LTPE system shows minimal resistance increase after 100 cycles, confirming stable interfacial characteristics due to effective side reaction suppression by the cathode electrolyte interphase (CEI) layers.

Long‐term cycling tests further validate system durability (Figure 7d,e,g; Figures S35 and S36, Supporting Information): The LTPE‐based cell delivers an initial discharge capacity of 174.6 mAh g^−1^ at 0.2C with 84.4% capacity retention after 100 cycles, far exceeding the 69.1% retention of PEO:LiTFSI. Fillers with precise pore size and specific functional groups are more conducive to improving cycling stability.^[^ ^55^, ^56^ ^]^ However, we believe that the capacity degradation in the LTPE battery is due to several factors that act synergistically. First, at lower current densities, deeper lithiation occurs, which may lead to more pronounced degradation of the lithium metal‐electrolyte interface. This is a common issue observed in polymer electrolytes. The deeper lithiation at lower currents could destabilize the interface, accelerating capacity fading.^[^ ^57^ ^]^ Particularly impressive is its 93.4% capacity retention over 2000 cycles at 2C (Figure 7e), with highly overlapping charge/discharge profiles (Figure 7f). La_0.994_Ta_0.06_OOH fillers promote TFSI^−^ decomposition through Lewis acid‐base interactions while inhibiting PEO backbone oxidation. Compared with existing PEO‐based systems (Figure 7h),^[^ ^58^, ^59^, ^60^, ^61^, ^62^, ^63^, ^64^, ^65^, ^66^ ^]^ this work demonstrates significant improvement in high‐rate performance. To validate practical applicability, we fabricated pouch cell batteries. These cells demonstrated an 80% capacity retention after 70 cycles at a 1C rate (Figure 7i). The initial charge/discharge profiles of the pouch cell are presented in Figure S37 (Supporting Information). Following formation cycling, the pouch cell retained an open‐circuit voltage of 3.4 V (Figure S38, Supporting Information). Critically, the batteries retained functionality and successfully powered a light‐emitting diode array even after being subjected to folding, bending, and cutting (Figure 7j), highlighting exceptional mechanical robustness and stable interfacial compatibility. Room‐temperature is critical for the cycling performance of solid‐state batteries; however, the ionic conductivity of PEO‐based solid electrolytes is typically low at room‐temperature, primarily because their conductivity is strongly dependent on the mobility of PEO molecular chains. To assess the practical viability of our electrolyte, we fabricated batteries using this electrolyte and conducted charge‐discharge tests, which revealed low capacity output. (Figure S39, Supporting Information) This suboptimal performance can be attributed to two key factors. On the one hand, the need to improve the conductivity of the filler material itself, and on the other hand, the need to improve the compatibility between the filler and PEO matrix. Furthermore, high interfacial resistance poses an additional significant barrier to overall performance. The challenges are exacerbated at low temperatures, poor compatibility between the PEO matrix and the filler disrupts the formation of continuous ion‐conducting pathways, which further limits the overall effectiveness of the electrolyte.

The morphological and chemical stability of electrode/electrolyte interfaces plays a pivotal role in determining the electrochemical performance of solid‐state batteries. To elucidate the structural evolution of CEI layers on LFP cathodes, we conducted systematic TEM characterization of electrodes cycled within 2.5–3.65 V. The TEM image of unrecycled LFP particles is shown in Figure S40 (Supporting Information). Cycling with the PEO:LiTFSI electrolyte resulted in a significantly thickened CEI layer (≈45 nm) on the LFP cathode (Figure S41a, Supporting Information). In contrast, the NWPE system reduced the CEI thickness to ≈13 nm; however, the resulting CEI layer displayed evident structural inhomogeneity and surface roughness (Figure S41b, Supporting Information). When highly dispersed cluster chains are used as electrolyte fillers, the blocking region and aggregation induced barrier of lithium ions are eliminated, forming a 3D network, realizing the high‐efficiency conductivity of lithium ions. At this time, the thickness and smoothness of the CEI film formed are further improved (Figure S41c, Supporting Information). Strikingly, the LTPE‐based electrolyte demonstrated exceptional interfacial compatibility with the LFP cathode, maintaining an ultra‐thin (< 8 nm) and remarkably uniform CEI layer even after 200 cycles (Figure S41d, Supporting Information). Elemental distribution mapping of LFP particles was conducted on both pristine and electrochemically cycled samples. Uniform elemental distributions were observed in all cases (Figure S42, Supporting Information), indicating good structural and interfacial stability. To further probe the chemical evolution of the cathode‐electrolyte interphase (CEI) in the LTPE system, XPS depth profiling was performed. The full spectrum before and after the cycle with LFP||LTPE||Li operated 60 °C is shown in Figure S43a (Supporting Information). The presence of C─O from organic lithium species and the C═O component from Li_2_CO_3_ indicates the formation of the CEI film. (Figure S43b, Supporting Information) Distinct characteristic peaks corresponding to Li_2_O (54.0 eV, Figure S43c, Supporting Information) and LiF (685.2 eV, Figure S43d, Supporting Information) were prominently identified.^[^ ^51^ ^]^ The superior interfacial stability and CEI characteristics achieved with the LTPE electrolyte surpass those reported for most state‐of‐the‐art solid‐state lithium metal batteries employing comparable cathodes, highlighting its significant potential for practical applications.

The SEI film plays a crucial role in the cycle life and safety of lithium metal batteries, as its chemical composition and structural morphology directly affect interface stability and lithium deposition behavior. To elucidate the differences in interfacial stability under different electrolyte systems, this paper systematically studied the composition and morphology evolution of SEI film on the surface of lithium metal anode after 50 cycles at a rate of 0.2C. The cycled Li anode was carefully extracted from the symmetric cell in an Ar‐filled glovebox. Following cycling in batteries employing LTPE, the SEI layer formed on the lithium metal anode surface exhibits no discernible cracks or particulate structures, demonstrating robust mechanical stability. In contrast, cycling within a PEO: LiTFSI electrolyte system resulted in the observation of SEI layers characterized by irregular particulate or porous morphologies. (Figure S44, Supporting Information) Studies indicate that such inhomogeneous and porous SEI layer morphologies typically compromise mechanical integrity and impair uniform ion transport. This degradation subsequently predisposes the system to lithium dendrite initiation and accelerates capacity fade. In‐depth XPS analysis of the SEI chemical composition at the Li metal anode/electrolyte interface after 50 cycles reveals that the LTPE system significantly reconstructs the SEI components. (Figure S45, Supporting Information) The peak at 286.5 eV is unambiguously assigned to the C‐S bond in TFSI^−^ (cross‐validated with the S 2p spectrum), while the intense peak at 290.7 eV corresponds to carbonate carbon in Li_2_CO_3_, whose intensity markedly increases in the modified interface. Displays a characteristic spin‐orbit splitting doublet (S 2p_3/2_: 169.1 eV, S 2p_1/2_: 170.3 eV), confirming the retention of undegraded TFSI^−^ with its ‐SO_2_‐ functional group.^[^ ^67^ ^]^ The bimodal distribution indicates enhanced LiF content in the SEI film formed after LTPE cycling. The altered intensity ratios of the three peaks at 56 eV (LiF), 55 eV (Li_2_CO_3_), and 54 eV (Li_2_O) conclusively demonstrate the formation of an inorganic‐rich SEI layer.^[^ ^68^ ^]^

## 3 Conclusion

This study demonstrates a rational atomic‐to‐nanoscale design strategy to address ion transport bottlenecks in PEO‐based composite electrolytes. By embedding highly dispersed Ta‐doped LaOOH cluster chains (La_0.94_Ta_0.96_OOH) into the polymer matrix, quantum confinement effects effectively suppress PEO crystallization and establish continuous 3D Li⁺ pathways, achieving a remarkable ionic conductivity of 0.65 mS cm^−1^ at 60 °C. Concurrently, Ta^5+^ substitution generates La vacancies that homogenize the electrostatic potential landscape, thereby reducing the energy barrier for Li⁺ migration and resulting in an elevated Li⁺ transference number of 0.47. These structural innovations synergistically mitigate interfacial resistance, as evidenced by ultra‐stable Li plating/stripping over 2000 h in symmetric cells and exceptional cyclability in full cells (93.4% capacity retention after 2000 cycles). Furthermore, the use of in situ multimodal c‐AFM provides direct evidence of temperature‐dependent electrolyte dynamics and spatially resolved ion transport heterogeneity, offering critical insights into interface evolution. This work not only establishes a universal framework for designing high‐performance CPEs through vacancy engineering but also highlights advanced characterization tools as indispensable guides for interface optimization. These findings pave the way for scalable fabrication of ASSLMBs while inspiring future exploration of dynamic interface phenomena in solid‐state energy systems.

## 4 Experimental Section

### Materials

Polyethylene oxide (PEO, Mw = 600 000), lanthanum(III) chloride (LaCl_3_·7H_2_O, 99.9%), tantalum chloride (TaCl_5_, 99.8%), and bis(trifluoromethane)sulfonimide (LiTFSI, 99%) were sourced from Shanghai Aladdin Biochemical Technology Co., Ltd. Acetonitrile (ACN, 99%), tetrahydrofuran (THF), oleic acid (AR), oleylamine (80–90%) and dichloromethane (DCM) were provided by Macklin Biochemical Technology Co., Ltd. Additionally, lithium iron phosphate (LiFePO_4_, 99%), 1‐methyl‐2‐pyrrolidone (NMP, 99%), polyvinylidene difluoride (PVDF, 99%)and Super P (99%) were acquired from Hefei Kejing Materials Technology Co., Ltd. All compounds were used as received without any further purification.

### Preparation of La_0.94_Ta_0.06_OOH

A precursor solution was prepared by dissolving lanthanum (III) chloride (LaCl_3_·7H_2_O) and tantalum chloride (TaCl_5_) in 1.8 mL of deionized water at different molar ratios, and then adding 21 mL of ethanol under ultrasonic treatment. A series of La_x_Ta_y_OOH samples was synthesized with a fixed total molar quantity (La + Ta) of 3.77 mm, The Ta doping level was varied at y = 0.02, 0.04, 0.06, and 0.08, which translates to La: Ta molar ratios of 98:2, 96:4, 94:6, and 92:8, respectively. The mixture was then introduced into a 50 mL autoclave containing oleylamine (7 mL) and oleic acid (3.5 mL) under continuous stirring. After maxing for 20 min, the sealed autoclave was heated at 160 °C for 8 h. The resulting La_0.94_Ta_0.06_OOH product was purified via sequential washing with ethanol and cyclohexane, followed by centrifugation to remove supernatants. The final product was stored in cyclohexane. For comparison, LaOOH was synthesized under identical conditions without TaCl_5_, using only LaCl_3_·7H_2_O (3.77 mm).

### Preparation of Composite Solid Electrolyte Membrane

A homogeneous solution was obtained by dissolving LiTFSI and PEO in acetonitrile (ACN) at an [EO]:[Li] molar ratio of 12:1 under 1.5 h stirring. Meanwhile, weighing different weights La_0.94_Ta_0.06_OOH chains were redispersed in a THF/DCM (1:1) mixture via ultrasonication for 1.5 h. The mass percentages of La_0.94_Ta_0.06_OOH relative to the total mass of PEO, LiTFSI, and La_0.94_Ta_0.06_OOH were 5 wt.% (LTPE‐5), 10 wt.%(LTPE‐10), 15 wt.%(LTPE‐15 or LTPE), and 20 wt.%(LTPE‐20), respectively.The transparent cluster suspension was then incorporated into the polymer electrolyte solution and stirred for 12 h. The resulting composite was cast into a PTFE mold and vacuum‐dried at 60 °C for 24 h. The freestanding electrolyte films were stored in an Ar‐filled glovebox for further use.

## Conflict of Interest

The authors declare no conflict of interest.

## Supporting information



Supporting Information

## Data Availability

The data that support the findings of this study are available from the corresponding author upon reasonable request.
