# Supplementary material for: A Sub‐1 Nm Cluster Chains‐enhanced Poly(ethylene oxide) Electrolyte for an All‐solid‐State Lithium Metal Battery with a Long Cycling Lifespan
Source: Adv Sci (Weinh). 2025 Nov 28;13(7):e16696. doi: 10.1002/advs.202516696 (PMC12866751; doi:10.1002/advs.202516696)
Supplement: Supplementary file 1 — Supporting Information [file ADVS-13-e16696-s001.docx]

Supporting Information

**A Sub-1 nm Cluster Chains-enhanced Poly(ethylene oxide) Electrolyte for an All-solid-state Lithium Metal Battery with a Long Cycling Lifespan.**

Shanshan Song, Fei He,* Yijun Gao, Yumeng Zhang, Junming Zhang, Tongjiao Yin, Qiqi Sun,* Zhiliang Liu,* Piaoping Yang

**EXPERIMENTAL SECTION**

*Fabrication of the composite cathode and cell assembly.* The LiFePO_4_ (LFP) composite cathode (1 C = 170 mAh g^−1^) was fabricated using a simple coating method. The LiFePO_4_, Super P, and PVDF powder were mixed in a solid weight ratio of 70:15:15 and stirred magnetically for one hour to ensure uniform dispersion. The resulting composite slurry was then coated onto an aluminum current collector and vacuum-dried at 80 °C for 10 hours. All preparation steps were conducted in an argon-filled glovebox with oxygen and moisture levels maintained below 0.01 ppm. No liquid additives were used during the cell assembly process. The mass of the active material (LFP) on the electrode sheet was calculated as follows: the total mass of the weighed electrode sheet was subtracted by the mass of the aluminum foil substrate, and the resulting difference was multiplied by the mass fraction of LFP in the cathode slurry (0.8). The mass of active material per unit area was obtained by dividing the total mass of the active material by the effective area of the electrode sheet. The average active material loading of the final electrode is approximately 1.0 mg cm⁻^2^, with a thickness of approximately 10 μm. **(Figure S46)**

*Electrochemical Performances of symmetric cells and full batteries.* The Li||CPEs||Li symmetric cells were assembled in an argon-filled glovebox (H_2_O < 0.01 ppm, O_2_ < 0.01 ppm) for constant current charging and discharging performance on the LAND battery test system at a current density of 0.1 mA cm^−2^ and capacity of 0.1 mAh cm^−2^. The cycle and rate performances at 60 °C of LiFePO_4_ LFP||CPEs||Li batteries were performed on the LAND battery test system with voltage ranges of 2.5 V-3.85 V. The solid-state full cells were assembled in CR2023-type button cells within controlled environments to prevent exposure to moisture and oxygen. Each cell was constructed with a cathode, an electrolyte membrane, and lithium metal.

*Materials characterizations.* The morphology of the filler and cathode materials was investigated using scanning electron microscopy (SEM, Hitachi, Japan) and transmission electron microscopy (TEM, JEM-2100F). X-ray diffraction (XRD) patterns were acquired using a Rigaku D/max-TTR-III diffraction equipment with Cu Kα radiation at 40 kV and 40 mA (λ = 0.15406 nm). X-ray photoelectron spectroscopy (XPS) was employed by PHI 5000 VersaProbe II (Ulvac-Phi, Japan) using Al Kα (1486.6 eV) excitation source. Differential scanning calorimetry (DSC) was performed between 23 °C and 80 °C using a NETZSCH STA 449 F3.

*Ionic conductivity calculated by electrochemical impedance spectroscopy.* Ionic conductivity (σ) of a CPE is converted from the resistance measured by electrochemical impedance spectroscopy (EIS) in a cell configuration of stainless symmetric electrodes from 0 °C to 60 °C. The calculation formula is as Eq. (1):

$$\sigma=\frac{l}{R_{b}S}$$

(1)

Where $\sigma$ (S cm^-1^) is the ionic conductivity of the e CPEs sample, $l$ (cm) is the thickness of the membrane-like CPE sample $R_{b}$ (Ω) is the bulk resistance measured by EIS, and $S$ (cm^2^) is the contact area between the e CPEs and the stainless steel.

*Calculate activation energies based on the ionic conductivity.*A lower energy barrier for ion migration within materials leads to a more rapid reaction rate. In the context of battery charging and discharging, reduced activation energy facilitates quicker reactions, ultimately enhancing both the power density and overall efficiency of the battery. The corresponding activation energies (Ea) were calculated from the Arrhenius Equation (2):

$$\sigma\left( T \right)=Aexp(-\frac{Ea}{RT})$$

(2)

in which $\sigma$ (S cm-1) is the ionic conductivity of the CPEs sample, A is the pre-exponential factor, R (J·(mol·K)^-1^) is gas constant and T (K) stands for the Kelvin temperature.

*Li-ion transference number calculation.* Li-ion transference number $t_{{Li}^{+}}$ is defined as the ratio of the current from cations to the total current, which is characterized by current detection under an applied voltage of 10 mV in the cell configuration of Li symmetric electrodes, with detailed calculation Eq. (4).

$$t_{{Li}^{+}}=\frac{I_{ss}\left( \Delta V-I_{0}R_{0} \right)}{I_{0}\left( \Delta V-I_{ss}R_{ss} \right)}$$

(4)

Where $t_{{Li}^{+}}$ is the Li-ion transference number of the CPEs sample, $I_{0}$ (A) and $I_{ss}$ (A) are the initial and steady-state current, respectively; $R_{0}$ (Ω) and $R_{ss}$ (Ω) are the initial and steady-state interfacial resistances measured by EIS, respectively; $\Delta V$ (V) is the applied voltage, which is set as 0.010 V.

*Quantum chemical calculations.*In the first-principles calculations using the CASTEP module, Density Functional Theory (DFT) was employed.^[1]^ The exchange-correlation effects were approximated using the generalized gradient approximation (GGA), as formulated in the Perdew-Burke-Ernzerhof (PBE) scheme. The calculations employed a kinetic energy cutoff of 450 eV for the plane-wave basis set. A 3 × 3 × 1 Monkhorst-Pack mesh k-point sampling was used to sample the Brillouin zone for the surface adsorption model. Convergence criteria were set at 1 × 10^−6^ eV/atom for energy and 0.02 eV/Å for maximum force. All structures were optimized until reaching their respective ground states, ensuring accuracy in the determination of electronic structures and surface properties. Reflecting realistic surface characteristics, a 2×3×2 supercell model of the LaOOH (0 1 1) surface was constructed. La_0.94_Ta_0.06_OOH model featured Ta^5+^ substitution for La^3+^ on the surface, along with surface La vacancies.

**Figure S1.** The optical photos of the LTPE membrane.

**Figure S2.** EPR spectra of La_x_Ta_y_OOH (y = 0.02, 0.04, 0.06, 0.8).

**Figure S3.** EIS curves of (a) La_0.98_Ta_0.02_OOH@PEO: LiTFSI, (b) La_0.96_Ta_0.04_OOH@PEO: LiTFSI, (c) La_0.94_Ta_0.06_OOH@PEO: LiTFSI,, and (d) La_0.92_Ta_0.08_OOH@PEO: LiTFSI at different temperatures.

**Figure S4.** Ionic conductivity of La_0.98_Ta_0.02_OOH@PEO: LiTFSI, La_0.96_Ta_0.04_OOH@PEO: LiTFSI, La_0.94_Ta_0.06_OOH@PEO: LiTFSI, and La_0.92_Ta_0.08_OOH@PEO: LiTFSI at different temperatures.

**Figure S5.** The XRD pattern of CW, LaOOH and La_0.94_Ta_0.06_OOH.

**Figure S6.** The Fourier transform infrared spectroscopy (FT-IR) spectra of NW, LaOOH and La_0.94_Ta_0.06_OOH.

**Figure S7.** TEM images from different regions of three separate batches of La_0.94_Ta_0.06_OOH products.

**Figure S8.** Small angle X-ray scattering spectra of La_0.94_Ta_0.06_OOH.

**Figure S9.** SEM images of LaOOH.

**Figure S10.** SEM images of La_0.94_Ta_0.06_OOH.

**Figure S11.** TEM images (a-b) and mapping image (c) of NW.

**Figure S12.** XPS spectra about the full spectrum, C 1s, La 3d, and O 1s of the LaOOH.

**Figure S13.** EIS curves of LTPE-5 and LTPE-20 at different temperatures.

**Figure S14.** Ionic conductivity of LTPE-5, LTPE-10, LTPE-15, and LTPE-20 at different temperatures.

**Figure S15.** Thermogravimetric analysis curve of the LTPE and PEO:LiTFSI, recorded under a nitrogen atmosphere with a heating rate of 5 °C min⁻¹ from 30 to 600 °C.

**Figure S16.** (a) SEM images of LPE. (b-d) The elemental mapping images of C, O, and La.

**Figure S17.** SEM images of LTPE and the elemental mapping images of C, O, La, and Ta.

**Figure S18.** (a) Side-view SEM image of the LTPE electrolyte. (b)The magnified inset of the LTPE electrolyte. (c) Elemental mapping images of C, O, La, Ta, overlay and the overlay superimposed on the SEM image.

**Figure S19.** The SEM image of the thickness of the CPEs.

**Figure S20.** Photographs of LNPE electrolyte at a) initial and b) final stretch state. The Stress-strain curves of PEO:LiTFSI, NWPE, LPE, and LTPE.

**Figure S21.** EIS curves of a) PEO: LiTFSI, b) NWPE, c) LPE and c) LTPE at different temperatures.

**Figure S22.** An image of the equivalent circuit diagram.

**Figure S23.** Current-time curves of Li symmetric cell, inserting is the corresponding EIS curves before and after polarization. a) PEO: LiTFSI, b) NWPE and c) LPE.

**Figure S24.** Infrared spectra of electrolyte membrane. (PEO: LiTFSI, NWPE, LPE, and LTPE)

**Figure S25.** GITT voltage profile and comparison of Li^+^ diffusion coefficients calculated from GITT analysis of lithiation and de-lithiation, respectively.

**Figure S26.** LSV curves of Li||Li symmetric cells from −0.3 to 0.3 V.

**Figure S27.** Comparative CV curves of a) PEO: LiTFSI and b) LTPE using different scan rates from 0.1 to 1.0 mV s^−1^.

**Figure S28.** EIS of LFP||LPE||Li batteries at different voltage charge (a) and discharge (d). The DRT transformation of EIS spectra during charge (b) and discharge (e). The DRT transform of 3D spectrum of EIS during charge (c) and discharge (f).

**Figure S29.** EIS of LFP||NWPE||Li batteries at different voltage charge (a) and discharge (d). The DRT transformation of EIS spectra during charge (b) and discharge (e). The DRT transform of 3D spectrum of EIS during charge (c) and discharge (f).

**Figure S30.** EIS of LFP||PEO:LiTFSI||Li batteries at different voltage charge (a) and discharge (d). The DRT transformation of EIS spectra during charge (b) and discharge (e). The DRT transform of 3D spectrum of EIS during charge (c) and discharge (f).

**Figure S31**. *In-situ* AFM characterization of PEO: LiTFSI electrolyte at (a_1_) 30 °C, (a_2_) 40 °C, (a_3_) and (a_4_) 50 °C. Corresponding 3D mode diagram. (b_1_-b_4_)

**Figure S32.** *In-situ* AFM characterization of LTPE electrolyte at (a_1_) 1 V, (a_2_) 2 V and (a_3_) 3 V at 30°C. c-AFM current under (b_1_) 1 V, (b_2_) 2 V and (b_3_) 3 V at 30°C. (c) c-AFM current curve at grain boundaries in Figure S21 b_1-3_.

**Figure S33.** *In-situ* AFM characterization of LTPE electrolyte at (e_1_) 1 V, (e_2_) 2 V and (e_3_) 3 V at 50°C.

**Figure S34.** (a) AFM morphology of c-AFM current under 3 V at 50 °C. (b) c-AFM current curve at grain boundaries in Figure S34(a).

**Figure S35.** Cycling performance of the LFP||CPEs||Li at current density of 0.2 C under 60 °C.

**Figure S36.** Cycling performance of the LFP||CPEs||Li at current density of 0.5 C under 60 °C.

**Figure S37.** The charge and discharge curves of all-solid-state pouch cells with the LTPE.

**Figure S38.** The open circuit voltage of the pouch cell of the LFP||LTPE||Li.

**Figure S39.** Cycling performance of the LFP||CPEs||Li at current density of 0.5 C under 25 °C.

**Figure S40.** TEM image of LFP particles.

**Figure S41.** High-resolution TEM images of LFP particles after cycling with different CPEs: (a) PEO: LiTFSI (b) NWPE, (c) LPE and (d) LTPE.

**Figure S42.** (a) HAADF-STEM image and elemental mapping of LFP particles. (b) HAADF-STEM image and elemental mapping of LFP particles after cycling with LTPE.

**Figure S43.** XPS on LFP surface based on LTPE before and after running 100 cycles: (g) full spectrum, (h) C 1s, (i) Li 1s and (j) F 1s.

**Figure S44.** SEM images of lithium contact surface and intersecting surface based on based on different electrolyte after 50 cycles at 0.2 C.

**Figure S45.** XPS spectra of SEI film of the interface between lithium and electrolyte after cycling: (a) C 1 s, (b) S 2p, (c) F 1 s, (d) Li 1 s.

**Figure S46.** SEM image of the thickness of the active material on the electrode.

**Table S1**. ICP data for the evaluation of La and Ta content of the La_x_Ta_y_OOH

| **Samples** | **(La/Ta) _ideal_** | **La (mg/mL)** | **Ta (mg/mL)** | **(La/Ta) _experiment_** | **error** |
| --- | --- | --- | --- | --- | --- |
| **La_0.98_Ta_0.02_OOH** | 37.11 | 0.7732 | 0.0214 | 36.13 | 2.64% |
| **La_0.96_Ta_0.04_OOH** | 17.95 | 0.7185 | 0.0418 | 17.18 | 4.29% |
| **La_0.94_Ta_0.06_OOH** | 11.52 | 0.6891 | 0.0603 | 11.42 | 0.87% |
| **La_0.92_Ta_0.08_OOH** | 8.32 | 0.6847 | 0.0779 | 8.7 | 4.57% |

**Table S2**. EXAFS fitting parameters at the La L-edge for various samples

| **Samples** | **Shell** | **CN^a^** | **R (Å)** | **σ^2^(Å^2^)** | **ΔE_0_(eV)^d^** | **R factor** |
| --- | --- | --- | --- | --- | --- | --- |
| **LaOOH** | La-O | 4 | 2.60±0.01 | 0.0192 | 16.86±0.33 | 0.0015 |
|  | La-La | 3 | 4.08±0.01 | 0.0016 |  |  |
|  | La-La | 4 | 5.04±0.01 | 0.0192 |  |  |
| **La_0.94_Ta_0.06_OOH** | La-O | 4.2 | 2.13±0.03 | 0.0282 | 7.00±0.51 | 0.0137 |
|  | La-La | 2.8 | 3.75±0.03 | 0.0197 |  |  |
|  | La-La | 4 | 4.73±0.03 | 0.0136 |  |  |

**Table S3. Comparison table of ion conductivity between this work and other studies.**

| **Samples** | **20 ^o^C (S cm^−1^)** | **60 ^o^C (S cm^−1^)** | **t_Li+_** | **ref** |
| --- | --- | --- | --- | --- |
| PEO/LiTFSI/MgI_2_ |  | 6.46×10^-4^ | 0.47 | *ACS Nano* 2025 [2] |
| CPE-1 M | 4.78×10^-5^(40 ^o^C) | 1.45×10^-3^ | 0.22 | *Chem. Eng. J.* 2025 [3] |
| PEO/UiO-66-NH_2_ | - | 3.6×10^-4^ | 0.58 | *Adv. Mater.* 2025 [4] |
| Bi-salt hc-PEO | - | 1.2 × 10^-4^ | 0.21 | *Adv. Mater.* 2025[5] |
| PEO/TOC@PEG | - | 2.32 × 10^-3^ | 0.47 | *Adv. Energy Mate*r. 2025[6] |
| PEO@DH |  | 2.02 ×10^-4^ | 0.34 | *Chem. Eng. J.* 2025[7] |
| CSE-10LHEO-f | 2.25×10^-4^(30 ^o^C) | 2.14 × 10^-3^ | 0.41 | *Adv. Funct. Mater.* 2024[8] |
| LCPE-60 | 6.74×10^-4^ | - | - | *Energy Storage Mater.* 2023[9] |
| PEO/LiTFSI/STO | 1.21×10^-5^ | 6.1×10^-4^ | 0.22 | *Adv. Funct. Mater.* 2025[10] |
| PEO@O-LZO | 1.63×10^-4^(40 ^o^C) | - | 0.59 | *Adv. Mater.* 2025[11] |
| PCPE-550 |  | 2.7×10^-4^ (50 °C) | 0.63 | *Angew. Chem. Int. Edit.* 2025[12] |
| LTPE | 1.1×10^-4^ | 6.5×10^-4^ | 0.47 | This work |

**Table S4.** Comparison of various PEO-based CPEs materials.

| Sample | Molecular weight of PEO (g mol^−1^) | Ionic conductivity | Temperature | Li^+^ transfer number | Charge/discharge performance of cells | Ref |
| --- | --- | --- | --- | --- | --- | --- |
| PEO/LiTFSI 3DPA-30 wt%Li-ILs@ZIF-8 | -- | 0.91×10^−3^ S cm^−1^ | 55 ^o^C | 0.40 | 1000 h at 0.1 mAcm^-2^ | [13] |
| PTFE@LLZO@PEO | 300,000 | 2.54 × 10^−4^ S cm^−1^ | 60 ^o^C | -- | 300 h at 0.1 mAcm^-2^ | [14] |
| Cu-MOF/P−Li | 600,000 | 9.58×10^−4^ S cm^−1^ | 60 ^o^C | 0.43 | 1080 h at 0.1 mAcm^-2^ | [15] |
| VCOF-1/PEO/LiTFSI/PVDF | 1,000,000 | 2.66 × 10^−4^ S cm^−1^ | 60 ^o^C | 0.38 | 300 h at 0.1 mAcm^-2^ | [16] |
| PEO-MOF-2 | 1,000,000 | 5.20×10^−4^ S cm^−1^ | 60 ^o^C | 0.36 | 350 h at 0.1 mAcm^-2^ | [17] |
| PEO/LiTFSI/3% NH_4_PF_6_@PA6 | 600,000 | ~2.0×10^−4^ S cm^−1^ | 60 ^o^C | -- | 2000 h at 0.1 mAcm^-2^ | [18] |
| Co-doped Li_4_Ti_5_O_12_-LiTFSI-PEO-10 | 1,000,000 | 1.09×10^−4^ S cm^−1^ | 60 ^o^C | 0.83 | 1800 h at 0.1 mAcm^-2^ | [19] |
| Single-ion conductor (ACA) in the PEO | 600,000 | 1.41 ×10^−4^ S cm^−1^ | 60 ^o^C | 0.52 | 900 h at 0.1 mAcm^-2^ | [20] |
| (GO-PSLi)-reinforced PEO | 600,000 | 2.2 ×10^−4^ S cm^−1^ | 60 ^o^C | 0.53 | 1100 h at 0.1 mAcm^-2^ | [21] |
| Metal-organic rotaxane framework in the PEO | -- | 4.02 ×10^−4^ S cm^−1^ | 60 ^o^C | 0.46 | 400 h at 0.05 mAcm^-2^ | [22] |
| MIL-53(Al)@PEO | 600,000 | 4.1 ×10^−4^ S cm^−1^ | 60 ^o^C | 0.40 | 1000 h at 0.1 mAcm^-2^ | [23] |
| LiGaO_2_, (AMIm-TFSI) | 600,000 | 2.08×10^−3^ S cm^−1^ | 60 ^o^C | 0.36 | 1000 h at 0.1 mAcm^-2^ | [24] |
| La_0.94_Ta_0.06_OOH@PEO: LITFSI | 600,000 | 6.5×10^−4^ S^−1^ | 60 ^o^C | 0.47 | 2000 h at 0.1 mAcm^-2^ | This work |

References

[1]	a) L. Bai, S. Ghiassinejad, J. Brassinne, Y. Fu, J. Wang, H. Yang, A. Vlad, A. Minoia, R Lazzaroni, J.F. Gohy, *ACS Appl. Mater. Interfaces* 2021, *13*, 44844-44859. b) K. M. Diederichsen, H. G. Buss, B. D. McCloskey, *Macromolecules* 2017, **50**, 3831-3840.

[2]	H. Yan, Q. Liu, W. Guo, C. Jiang, Y. Song, F. Zhou, C. Bao, B. Li, *ACS Nano* 2025, DOI: 10.1021/acsnano.5c12885.

[3]	N. Zhang, S.-H. Tian, X.-Q. Chen, P.-F. Wang, Z.-L. Liu, J. Shu, T.-F. Yi, *Chem. Eng. J.* 2025, **524**, 169591.

[4]	L. Feng, Z. Z. Shen, Q. Yang, J. Zhang, S. Chen, *Adv. Mater.* 2025, e12360.

[5]	Z. Kong, Y. Wu, J. F. Wu, X. Liang, Y. Li, Y. Lin, J. Liu, *Adv. Mater.* 2025, e14236.

[6]	Z. Yang, Z. Yang, J. Liu, G. Ye, S. Chen, R. Li, H. Zhang, T. Huang, Q. Pang, H. Liu, *Adv. Energy Mate*r. 2025, e03760.

[7]	X. Zhu, Z. Wang, Z. Xu, J. Li, H. Yang, B. Luo, B. Guo, K. Wang, *Chem. Eng. J.* 2025, **513**, 162812.

[8]	W. Liu, J. Liu, Z. Yang, M. Liu, S. Sang, H. Liu, *Adv. Funct. Mater.* 2024, **35**, 2419095.

[9]	X. Zhang, C. Fu, S. Cheng, C. Zhang, L. Zhang, M. Jiang, J. Wang, Y. Ma, P. Zuo, C. Du, Y. Gao, G. Yin, H. Huo, *Energy Storage Mater.* 2023, **56**, 121.

[10]	Y. Guo, M. Zhang, Z. Ge, Z. Fang, Z. Xu, J. Wu, M. Wu, *Adv. Funct. Mater.* 2025, **35**, 2419998.

[11]	Y. Wang, T. Fang, C. Wang, S. Wang, K. Yang, J. Biao, D. Li, D. Yang, Y. B. He, Y. Xia, *Adv. Mater.* 2025, **37**, 202505209.

[12]	Y. Ren, S. Chen, M. Odziomek, J. Guo, P. Xu, H. Xie, Z. Tian, M. Antonietti, T. Liu, *Angew. Chem. Int. Edit.* 2025, **64**, e202422169.

[13]	X. Zheng, J. Wu, J. Chen, X. Wang, Z. Yang, *J. Energy Chemistry* 2022, **71**, 174.

[14]	Z.-C. Li, T.-Y. Li, Y.-R. Deng, W.-H. Tang, X.-D. Wang, J.-L. Yang, Q. Liu, L. Zhang, Q. Wang, R.-P. Liu, *Rare Met.* 2022, **41**, 2834.

[15]	J. Wu, Y. Ma, H. Zhang, H. Xie, J. Hu, C. Shi, B. Chen, C. He, N. Zhao, *ACS Appl. Mater. Interfaces* 2024, **16**, 16351.

[16]	J. Yang, C. Lin, Y. Wang, Y. Xu, D. T. Pham, X. Meng, K. V. Tran, S. Cao, N. Kardjilov, A. Hilger, J. D. Epping, I. Manke, A. Thomas, Y. Lu, *J. Mater. Chem. A* 2024, **12**, 1694.

[17]	G. Lu, H. Wei, C. Shen, F. Zhou, M. Zhang, Y. Chen, H. Jin, J. Li, G. Chen, J. Wang, S. Wang, *ACS Appl. Mater. Interfaces* 2022, **14**, 45476.

[18]	L. Han, Y. Liu, C. Liao, Y. Zhao, Y. Cao, Y. Kan, J. Zhu, Y. Hu, *Nano Energy* 2023, **112**, 108448.

[19]	Z. Guo, S. Liao, F. Li, L. Shi, B. Su, J. Mu, Y. Wang, Z. Huang, F. Xu, C. Guan, *Chem. Eng. J.* 2024, **496**, 154163.

[20]	Z. Hu, F. Ji, Y. Zhang, W. Guo, X. Jing, W. Bao, J. Qin, S. Huo, S. Li, Y. Zhang, W. Fan, H. Cheng, *Chem. Eng. J.* 2023*,* **468***, 143857*.

[21]	Z. Hu, W. Bao, Y. Zhang, X. Jiang, J. Chen, Y. Zhang, S. Huo, J. Zhang, J. Qin, S. Li, W. Guo, H. Cheng, *Chem. Eng. J.* 2023, **472**, 144932.

[22]	Y. Li, Y. Xu, X. Han, D. Han, X. Wu, X. Wang, Z. Su, Chin. Chem. Lett. 2024, **35***,* 109189*.*

[23]	L. Wang, L. Xie, L. Dong, Z. Wang, L. Li, E. Shangguan, J. Li, S. Gao, *J. Colloid Interface Sci.* 2024, **657**, 632-643.

[24]	X. Luo, X. Xu, F. Li, S. Ji, W. Fan, J. Zhao, J. Liu, Y. Huo, *J. Power Sources* 2024, **618***,* 235165.
